# Vascular-related biomarkers in psychosis: a systematic review and meta-analysis

**DOI:** 10.3389/fpsyt.2023.1241422

**Published:** 2023-08-25

**Authors:** Xiaojun Li, Shuang Hu, Pozi Liu

**Affiliations:** ^1^Tsinghua University School of Medicine, Beijing, China; ^2^Shanghai Mental Health Center, Shanghai Jiaotong University School of Medicine, Shanghai, China; ^3^Department of Psychiatry, Beijing Yuquan Hospital, Tsinghua University, Beijing, China

**Keywords:** psychotic disorders, vascular dysfunction, blood–brain barrier, neurovascular unit, meta-analysis

## Abstract

**Background:**

While the molecular underpinnings of vascular dysfunction in psychosis are under active investigation, their implications remain unclear due to inconsistent and sometimes sparse observations. We conducted a comprehensive meta-analysis to critically assess the alterations of vascular-related molecules in the cerebrospinal fluid (CSF) and blood of patients with psychotic disorders compared with healthy individuals.

**Methods:**

Databases were searched from inception to February 23, 2023. Meta-analyses were performed using a random-effects model. Meta-regression and subgroup analyses were conducted to assess the effects of clinical correlates.

**Results:**

We identified 93 eligible studies with 30 biomarkers investigated in the CSF and/or blood. Among the biomarkers examined, psychotic disorders were associated with elevated CSF-to-serum albumin ratio (standardized mean difference [SMD], 0.69; 95% confidence interval [CI], 0.35–1.02); blood S100B (SMD, 0.88; 95% CI, 0.59–1.17), matrix metalloproteinase-9 (MMP-9; SMD, 0.66; 95% CI, 0.46–0.86), and zonulin (SMD, 1.17; 95% CI, 0.04–2.30). The blood levels of S100B, MMP-9, nerve growth factor (NGF), vascular endothelial growth factor (VEGF), intercellular adhesion molecule 1 (ICAM-1), and vascular adhesion molecule 1 (VCAM-1) were altered in patient subgroups differing in demographic and clinical characteristics. Blood S100B level was positively correlated with age and duration of illness. Substantial between-study heterogeneity was observed in most molecules.

**Conclusion:**

The alterations in certain vascular-related fluid markers in psychotic disorders suggest disturbances in normal vascular structures and functions. However, not all molecules examined displayed clear evidence of changes. While potential impacts of clinical factors, including the administered treatment, were identified, the exploration remained limited. Further studies are needed to investigate the diverse patterns of expression, and understand how these abnormalities reflect the pathophysiology of psychosis and the impact of clinical factors.

## 1. Introduction

Growing evidence has revealed the presence of both peripheral and central vascular dysfunction in psychotic disorders. Meta-analyses have shown that patients with schizophrenia carry a greater risk of cardiovascular diseases even in their first episodes prior to antipsychotic exposure (1–3), with this risk being even higher in chronic schizophrenia (4). Cardiovascular risk factors are further linked to cognitive impairment (5) and contribute to mortality (6, 7). In the central nervous system (CNS), postmortem studies have revealed direct evidence of destruction of the blood–brain barrier (BBB) and neurovascular unit (NVU) (8, 9). Functionally, positron emission tomography (PET) studies have indicated increased activity of the efflux transporter P-glycoprotein in multiple brain regions, suggesting altered BBB permeability (10). Moreover, the NVU plays a key role in neurovascular coupling (NVC), in which cerebral blood flow is regulated in response to changes in energy utilization and neural activity. NVC deficits in the frontotemporal regions have been consistently observed in fMRI studies in patients with first-episode psychosis (FEP) (11). The strength of coupling is closely modulated by the density, morphology, and capacity of dilation of the vessels (12). Notably, patients with schizophrenia exhibit thinner neurites but similar capillary diameters compared to healthy controls, implying a neurite-vasculature mismatch (13, 14). Furthermore, reduced grey matter blood flow across multiple brain areas has been observed in patients with schizophrenia (15). Correlation exists between the abnormal cerebral blood flow and symptom severity (16), both of which improved with aripiprazole treatment (17).

Apart from neuropathology and neuroimaging studies, much effort has been devoted to investigating the relevant fluid biomarkers in psychotic disorders. The goal has been to obtain easily accessible information regarding the mechanisms, diagnosis, and prognosis of these disorders (9, 18). However, conflicting observations exist, and the implications of vascular-related fluid biomarkers remain a subject of debate. To date, most meta-analyses in this field focused only on specific molecules. In addition, many earlier meta-analyses included non-peer-reviewed studies or those involving participants with comorbid conditions known to affect vascular functions, such as cardiovascular diseases and cannabis use, which can introduce bias and limit the generalizability of the findings.

Here, we aim to provide a comprehensive meta-analysis to further evaluate the alterations in vascular-related fluid molecules in psychotic disorders, along with their relations with demographic and clinical characteristics.

## 2. Methods and materials

This study adhered to the Preferred Reporting Items for Systematic Reviews and Meta-analyses (PRISMA) reporting guidelines (Supplementary Table S1) (19). The study protocol was registered in PROSPERO (CRD42023398731). Two authors (XL and SH) independently performed the literature search, study inclusion, and bias assessment. One author (XL) extracted the data, which was then examined by another author (SH). When discrepancies occurred, consensus was reached either through discussion between the two authors or by consulting the third author (PL).

### 2.1. Search strategy

We searched Embase, MEDLINE, PsycINFO, and Web of Science from database inception to February 23, 2023 as well as the reference lists of the retrieved studies. The search term is presented in Supplementary Table S2. Deduplication was performed using the Bramer method (20).

### 2.2. Selection criteria

Studies were included if they fulfilled the following criteria: (1) peer-reviewed and published in English; (2) quantified vascular-related biomarkers in the CSF or blood from living patients and healthy controls; (3) patients were diagnosed with schizophrenia, schizoaffective disorder, schizophreniform disorder, delusional disorder, or FEP according to a recognized diagnostic system (e.g., Diagnostic and Statistical Manual of Mental Disorders [DSM], International Classification of Diseases [ICD], or Research Diagnostic Criteria [RDC]).

Studies were excluded if they (1) are reviews, letters to the editor, correspondence, or published in conferences; (2) contained participants with other psychiatric disorders, substance use, neurological disorders, cardiovascular diseases, or inflammatory diseases; (3) contained controls with personal or family history of psychiatric disorders.

For studies containing overlapping participants, we included only studies that first reported the most comprehensive information.

### 2.3. Data extraction

Data extracted include the number, age, gender, body mass index (BMI), and smoking status of all participants; diagnosis, diagnostic tool, age at onset, duration of illness, treatment status (i.e., drug-naïve, unmedicated, medicated, and post-ECT) and duration, the types and doses of medications and symptom severity of the patients; concentrations of the biomarkers, sample origins, and measurement methods of the biomarkers. For longitudinal studies involving different treatment statuses, both baseline and follow-up data were extracted. In cases of missing data, data reported as summary estimates other than arithmetic means and standard deviations (SDs), or uncertainty, authors of the studies were contacted to provide information.

When the requested information was not received, we extracted the data using WebPlotDigitizer, version 4.6 (Ankit Rohatgi, CA, USA) if it was reported in graphs in the articles. Medians, ranges, and interquartile ranges were converted to means and SDs using validated methods (21, 22). When the concentrations of a biomarker were reported in arithmetic and geometric scales in different studies, statistics in the scale less frequently reported in the included studies were converted to the other scale using the Higgins approach (23).

### 2.4. Bias assessment

Bias assessment was performed using an adapted version of the Newcastle-Ottawa Scale (NOS; Supplementary Table S3) (24). Assessment of the item “non-response rate” was not applicable, thus the maximum total score on NOS was 8. Small-study effects were assessed with contour-enhanced funnel plots (25) and the Egger test (26) for biomarkers measured in 10 or more studies.

### 2.5. Statistical analyses

Our primary outcome was the differences in biomarker concentration between patients and healthy controls. Secondary outcomes were the associations between biomarkers and demographic and clinical characteristics. Meta-analyses were performed using Stata/SE, version 17.0 (StataCorp LLC).

Heterogeneity between studies was assessed using the Cochran’s *Q* test (27). Level of significance was set at *p* < 0.10. Degree of heterogeneity was quantified using the *I*^2^ metric (28), with metrics of 25, 50, and 75% considered as indicating low, moderate, and high heterogeneity, respectively (29). Our analyses showed high heterogeneity among the majority of studies, thus random-effects model (30) was used to calculate the standardized mean differences (SMDs) as the effect size. SMDs were bias-corrected using Hedges’ *g* (28). We consider an effect size of 0.2, 0.5, and 0.8 as indicating a low, moderate, and large effect, respectively. Level of significance was set at *p* < 0.05. In cases of longitudinal studies where biomarkers were measured under different treatment statuses, we used the measurement under the status that most patients tested for the biomarker were under.

Outlier studies were detected using the Galbraith plot (31). Sensitivity analyses were performed by removing the outliers and recomputing the overall significance.

For biomarkers quantified in 5 or more studies, we conducted subgroup analyses to assess the effect of illness stages (i.e., FEP and non-FEP), treatment status (i.e., drug-naïve, unmedicated, medicated, and post-ECT), and sample origins (i.e., plasma and serum). For biomarkers quantified in 10 or more studies, meta-regression was conducted to investigate the effects of demographic and clinical characteristics if 10 or more data points were available. For biomarkers quantified in only one study, findings of the studies were qualitatively summarized.

## 3. Results

**Figure 1:**
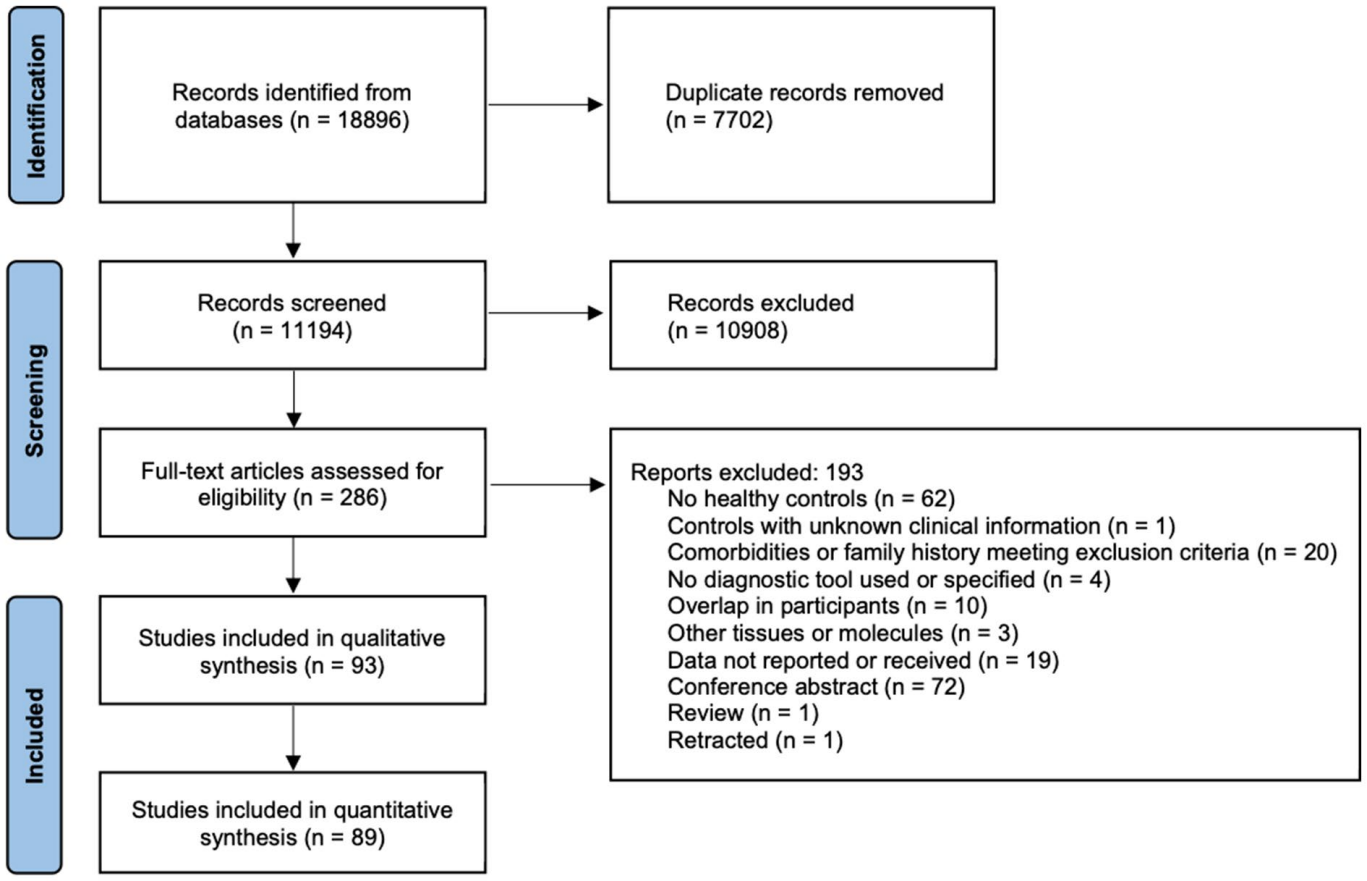
PRISMA 2020 flow diagram. The literature search, and the screening and inclusion of eligible studies following the PRISMA reporting guidelines are shown.

**Figure 2:**
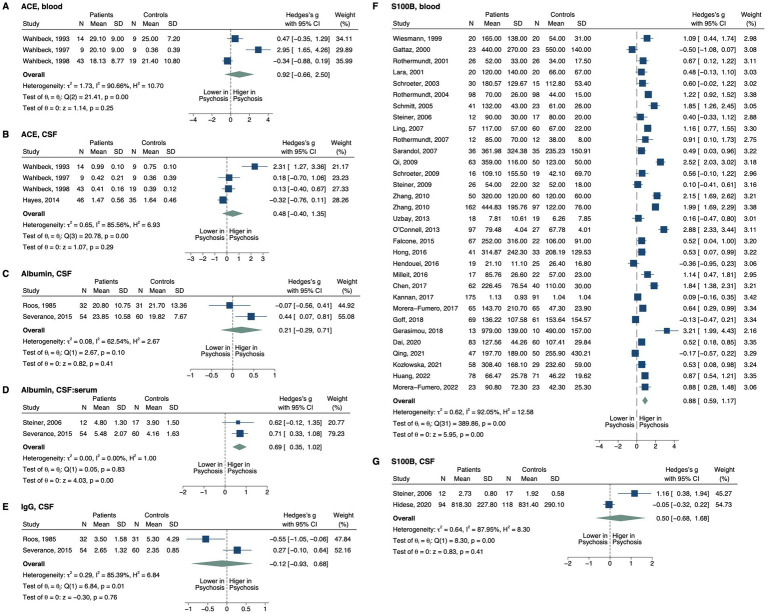
Forest plots of the markers of BBB permeability measured in ≥2 studies. Forest plots of **(A)** blood ACE, **(B)** CSF ACE, **(C)** CSF albumin, **(D)** CSF:serum albumin, **(E)** CSF IgG, **(F)** blood S100B, and **(G)** CSF S100B. The sizes of the squares are proportional to the weights calculated from random-effects analysis. Horizontal lines represent 95% CIs. The diamond denotes the overall SMD. The vertical dashed line represents the line of no difference between patients and controls.

**Figure 3:**
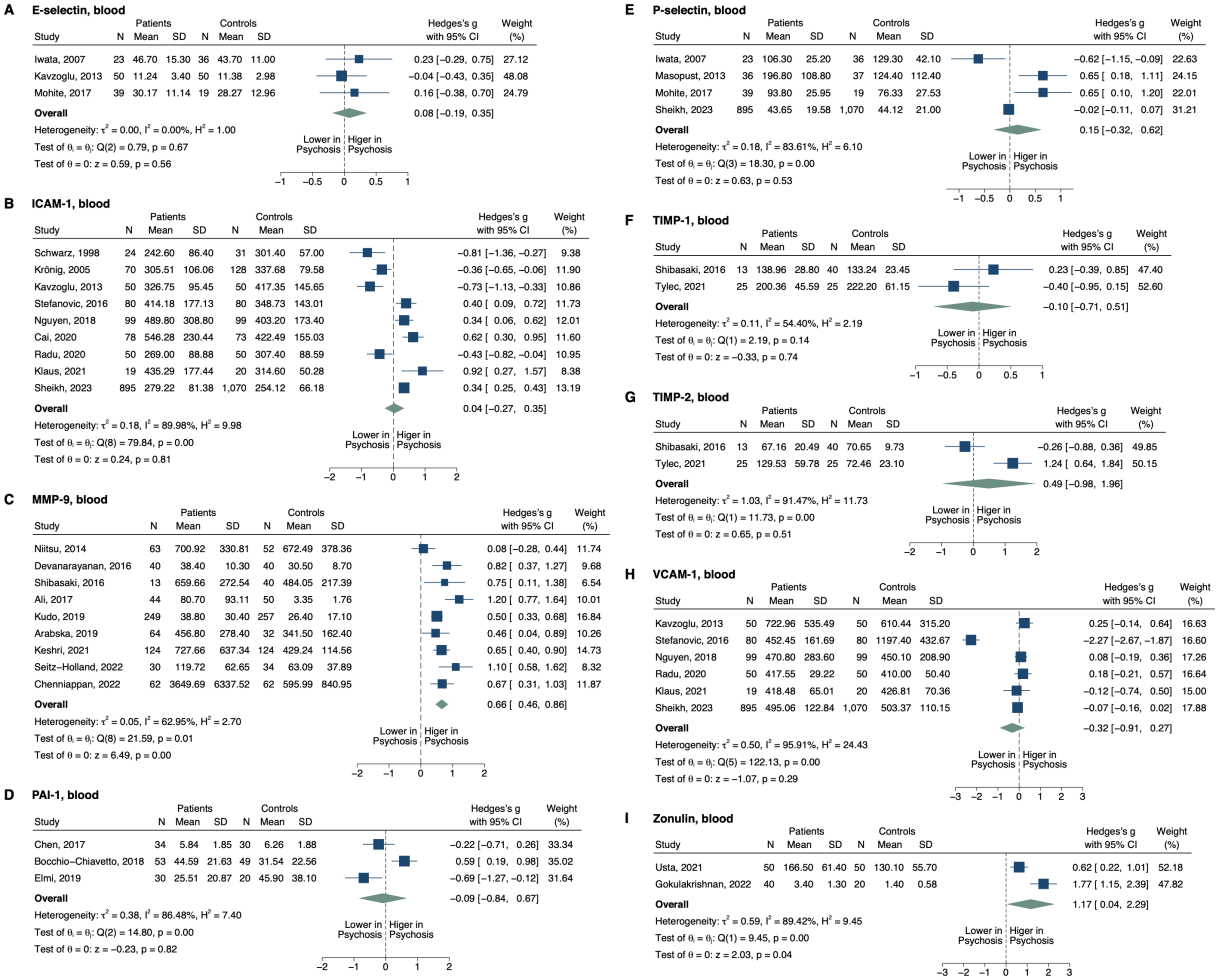
Forest plots of tight junction proteins and modifiers measured in ≥2 studies. Forest plots of blood **(A)** E-selectin, **(B)** ICAM-1, **(C)** MMP-9, **(D)** PAI-1, **(E)** P-selectin, **(F)** TIMP-1, **(G)** TIMP-2, **(H)** VCAM-1, and **(I)** zonulin. The sizes of the squares are proportional to the weights calculated from random-effects analysis. Horizontal lines represent 95% CIs. The diamond denotes the overall SMD. The vertical dashed line represents the line of no difference between patients and controls.

**Figure 4:**
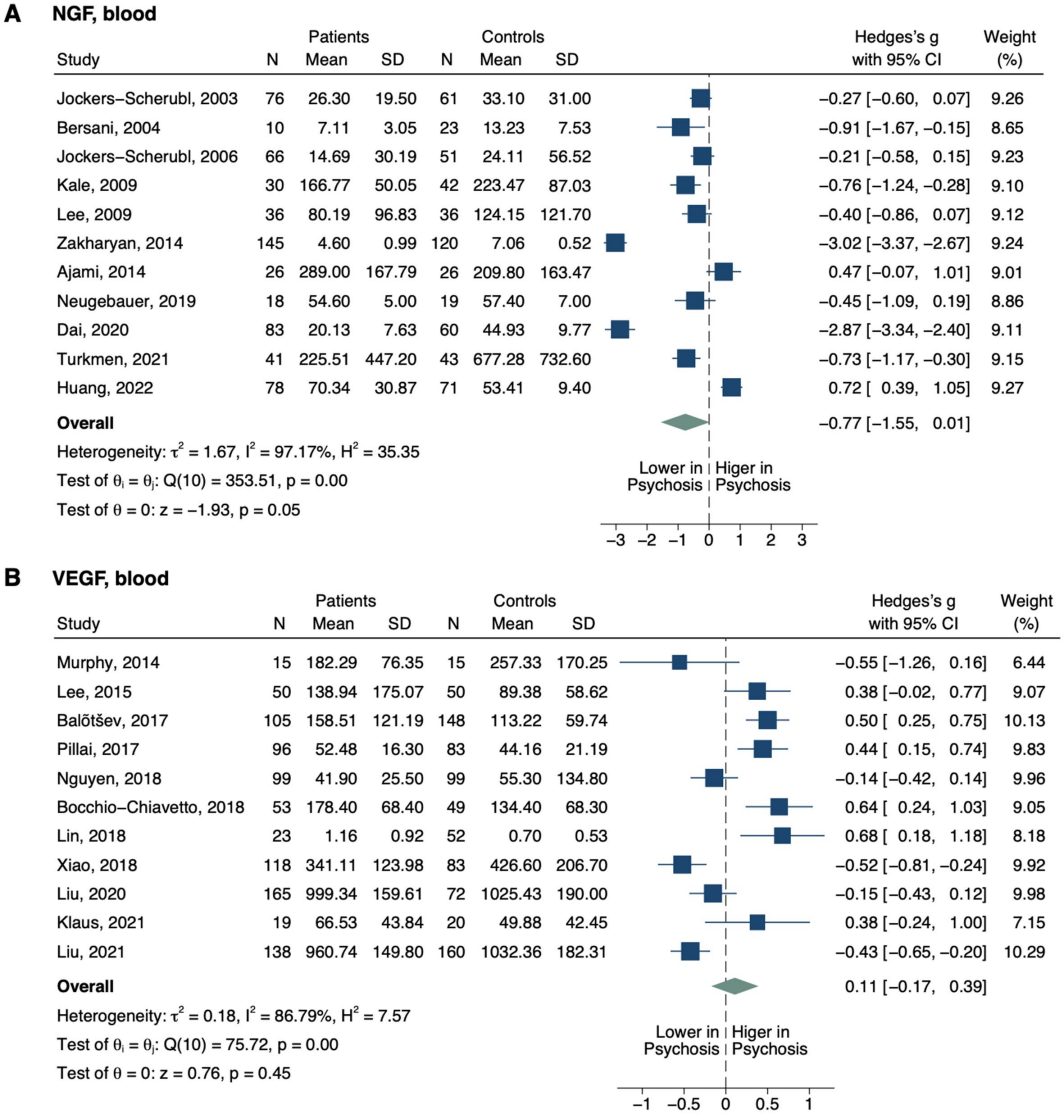
Forest plots of angiogenic neurotrophins measured in ≥2 studies. Forest plots of blood **(A)** NGF and **(B)** VEGF. The sizes of the squares are proportional to the weights calculated from random-effects analysis. Horizontal lines represent 95% CIs. The diamond denotes the overall SMD. The vertical dashed line represents the line of no difference between patients and controls.

**Figure 5:**
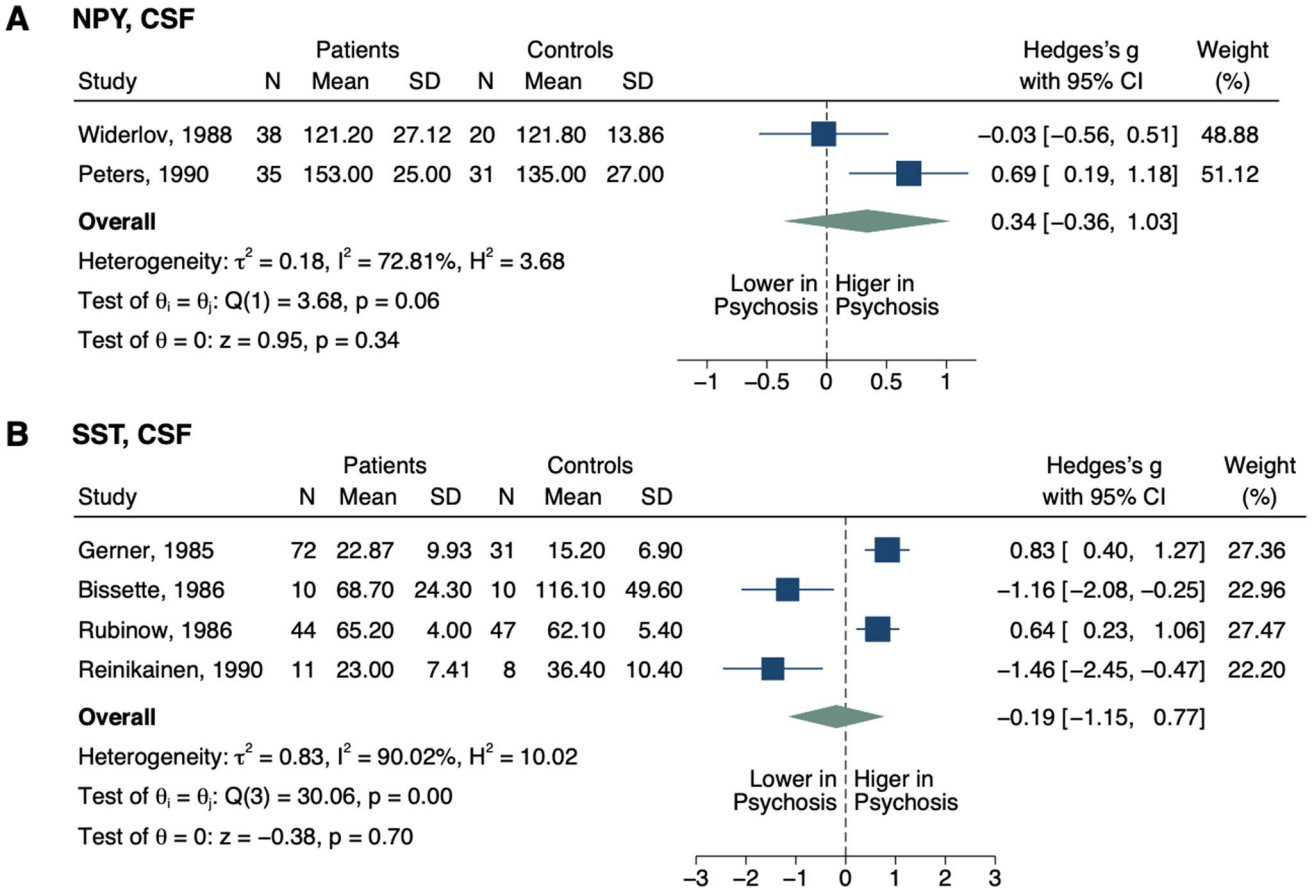
Forest plots of neuropeptides involved in neurovascular coupling measured in ≥2 studies. Forest plots of CSF **(A)** NPY and **(B)** SST. The sizes of the squares are proportional to the weights calculated from random-effects analysis. Horizontal lines represent 95% CIs. The diamond denotes the overall SMD. The vertical dashed line represents the line of no difference between patients and controls.

The literature search identified 11,194 studies after deduplication, of which 93 were included in the final synthesis (Figure 1; Supplementary Tables S4, S5). Thirty biomarkers in the CSF and/or blood were identified, of which 17 were quantified in two or more studies (Table 1; Figures 2–5). Findings of the remaining 13 biomarkers quantified in only one study are summarized in Supplementary Table S6.

**Table 1:** Effect sizes of molecules examined in ≥2 studies.

Molecule	Tissue	Number			Effect size			Heterogeneity	
		Studies	Patients	Controls	Hedges’ *g*	95% CI	*p*	*Q*	*p*^a^
ACE	CSF	4	112	72	0.48	[−0.40, 1.35]	0.285	20.78	**<0.001**
ACE	Serum	3	66	37	0.92	[−0.66, 2.50]	0.254	21.41	**<0.001**
Albumin	CSF	2	86	91	0.21	[−0.29, 0.71]	0.413	2.67	0.102
Albumin	CSF:serum	2	66	77	0.69	[0.35, 1.02]	**<0.001**	0.05	0.825
E-selectin	Blood	3	112	105	0.08	[−0.19, 0.35]	0.555	0.79	0.672
ICAM-1	Blood	9	1,365	1,601	0.04	[−0.27, 0.35]	0.809	79.84	**<0.001**
IgG	CSF	2	86	91	−0.12	[−0.93, 0.68]	0.765	6.84	**0.009**
MMP-9	Blood	9	689	691	0.66	[0.46, 0.86]	**<0.001**	21.59	**0.006**
NGF	Blood	11	609	552	−0.77	[−1.55, 0.01]	0.054	353.51	**<0.001**
NPY	CSF	2	73	51	0.34	[−0.36, 1.03]	0.341	3.68	**0.055**
P-selectin	Blood	4	993	1,162	0.15	[−0.32, 0.62]	0.529	18.30	**<0.001**
PAI-1	Blood	3	117	99	−0.09	[−0.84, 0.67]	0.819	14.80	**0.001**
S100B	CSF	2	106	135	0.50	[−0.68, 1.68]	0.408	8.30	**0.004**
S100B	Blood	32	1,624	1,255	0.88	[0.59, 1.17]	**<0.001**	389.86	**<0.001**
SST	CSF	4	137	96	−0.19	[−1.15, 0.77]	0.700	30.06	**<0.001**
TIMP-1	Serum	2	38	65	−0.10	[−0.71, 0.51]	0.744	2.19	0.139
TIMP-2	Serum	2	38	65	0.49	[−0.98, 1.96]	0.514	11.73	**<0.001**
VCAM-1	Blood	6	1,193	1,369	−0.32	[−0.91, 0.27]	0.285	122.13	**<0.001**
VEGF	Blood	11	881	831	0.11	[−0.17, 0.39]	0.447	75.72	**<0.001**
Zonulin	Blood	2	90	70	1.17	[0.04, 2.30]	**0.043**	9.45	**0.002**

ACE, angiotensin-converting enzyme; CI, confidence interval; CSF, cerebrospinal fluid; ICAM-1, intercellular adhesion molecule 1; IgG, immunoglobulin G; MMP-9, matrix metalloproteinase-9; NGF, nerve growth factor; NPY, neuropeptide Y; PAI-1, plasminogen activator inhibitor-1; SST, somatostatin; TIMP, tissue inhibitor of matrix metalloproteinase; VCAM-1, vascular cell adhesion molecule 1; VEGF, vascular endothelial growth factor.

a Statistical significance at 0.1 level.

### 3.1. Bias assessment

The average NOS score of the studies was 4.90 ± 1.68 (mean ± SD), with 17 (18.09%) studies scoring less than 4, 76 (80.85%) scoring at least 4, and 5 (5.32%) scoring 8 (Supplementary Table S7). The majority of studies had risks of bias in case definition (54.26%) and ascertainment of exposure (79.79%). Thirty-two (34.04%) studies did not state to have matched or adjusted for any confounding factors. Others had risks of bias in case representativeness (24.47%), control selection (44.68%), and control definition (24.47%).

Contour-enhanced funnel plots of NGF, S100B, and VEGF blood levels are shown in Supplementary Figure S1. No small-study effects were detected for these 3 biomarkers (Egger test: NGF, *p* = 0.958; S100B, *p* = 0.167; VEGF, *p* = 0.653; Supplementary Table S8).

### 3.2. Blood–brain barrier

For markers evaluating BBB permeability (Figure 2), CSF-to-serum albumin ratio was significantly elevated in patients compared to controls (SMD, 0.69; 95% CI, 0.35–1.02; *p* < 0.001) (32, 33), whereas CSF albumin did not display a significant difference (SMD, 0.21; 95% CI, −0.29–0.71; *p* = 0.413) (33, 34). Blood S100B was also elevated in psychosis, with a large effect size (SMD, 0.88; 95% CI, 0.59–1.17; *p* < 0.001) (32, 35–65), which remained significant after excluding 3 outlier studies (Supplementary Figure S2; SMD, 0.70; 95% CI, 0.44–0.96; *p* < 0.001) (46, 55, 56). In contrast, CSF S100B did not differ between groups (SMD, 0.50; 95% CI, −0.68–1.68; *p* = 0.408) (32, 66). The increase in blood S100B was detected in patients across 3 treatment statuses—drug-naïve, unmedicated and medicated (Table 2). This pattern was consistently observed in serum (SMD, 1.06; 95% CI, 0.72–1.40; *p* < 0.001) and patients with non-FEP (SMD, 0.93; 95% CI, 0.61–1.24; *p* < 0.001), rather than in plasma (SMD, 0.35; 95% CI, −0.09–0.78; *p* = 0.118) or patients with FEP (SMD, 0.53; 95% CI, −0.09–1.16; *p* = 0.096). No significant differences were found for CSF or blood angiotensin-converting enzyme (ACE) (67–70), or CSF immunoglobulin G (IgG) levels (33, 34).

**Table 2:** Subgroup analysis of molecules examined in ≥5 studies.

Subgroup	Number			Effect size			Heterogeneity		
	Studies	Patients	Controls	Hedges’ *g*	95% CI	*P*	*I*^2^ (%)	*Q*	*P*^a^
*Blood ICAM-1*									
Drug-naïve	2	100	100	−0.81	[−1.10, −0.53]	**<0.001**	0.00	0.33	0.567
Unmedicated	2	150	208	0.02	[−0.72, 0.77]	0.954	91.80	12.20	**<0.001**
Medicated	5	290	303	0.12	[−0.39, 0.63]	0.656	89.03	36.45	**<0.001**
Non-FEP	6	1,241	1,470	0.33	[0.07, 0.59]	**0.012**	81.97	27.73	**<0.001**
FEP	2	100	100	−0.58	[−0.87, −0.28]	**<0.001**	8.91	1.10	0.295
Plasma	5	1,141	1,312	0.28	[−0.09, 0.64]	0.140	87.90	33.06	**<0.001**
Serum	4	224	289	−0.27	[−0.77, 0.22]	0.281	86.12	21.62	**<0.001**
*Blood MMP-9*									
Unmedicated	1	44	50	1.21	[0.77, 1.64]	**<0.001**	/	/	/
Medicated	4	168	188	0.59	[0.16, 1.02]	**0.007**	72.44	10.88	**0.012**
Post-ECT	1	13	40	0.09	[−0.53, 0.71]	0.777	/	/	/
Plasma	2	279	291	0.75	[0.18, 1.32]	**0.010**	77.60	4.46	**0.035**
Serum	7	410	400	0.65	[0.40, 0.90]	**<0.001**	64.36	16.84	**0.010**
*Blood NGF*									
Drug-naïve	5	233	334	−2.16	[−4.02, −0.31]	**0.022**	98.58	281.99	**<0.001**
Unmedicated	3	114	120	0.23	[−0.62, 1.08]	0.601	87.16	15.58	**<0.001**
Medicated	6	307	295	−0.72	[−1.77, 0.34]	0.182	96.98	165.72	**<0.001**
Non-FEP	8	351	330	−0.19	[−0.59, 0.21]	0.340	84.32	44.65	**<0.001**
FEP	2	113	102	−1.82	[−3.89, 0.26]	0.086	97.38	38.13	**<0.001**
Plasma	4	221	221	−1.28	[−2.69, 0.13]	0.075	97.09	103.07	**<0.001**
Serum	7	388	331	−0.47	[−1.31, 0.36]	0.263	96.35	164.24	**<0.001**
*Blood S100B*									
Drug-naïve	4	223	217	1.45	[0.01, 2.88]	**0.048**	97.49	119.31	**<0.001**
Unmedicated	8	360	362	0.90	[0.55, 1.25]	**<0.001**	78.03	31.87	**<0.001**
Medicated	22	1,043	906	1.04	[0.66, 1.43]	**<0.001**	93.05	302.34	**<0.001**
Non-FEP	26	1,383	1,035	0.93	[0.61, 1.24]	**<0.001**	91.91	309.16	**<0.001**
FEP	5	224	198	0.53	[−0.09, 1.16]	0.096	88.22	33.96	**<0.001**
Plasma	8	301	292	0.35	[−0.09, 0.78]	0.118	84.84	46.18	**<0.001**
Serum	24	1,323	963	1.06	[0.72, 1.40]	**<0.001**	92.21	295.26	**<0.001**
*Blood VCAM-1*									
Drug-naïve	2	100	100	0.55	[−0.04, 1.15]	0.067	77.29	4.40	**0.036**
Unmedicated	1	80	80	−2.27	[−2.67, −1.87]	**<0.001**	/	/	/
Medicated	3	188	199	−0.52	[−1.63, 0.59]	0.357	96.07	50.88	**<0.001**
Non-FEP	4	1,093	1,269	−0.59	[−1.46, 0.28]	0.182	97.41	115.69	**<0.001**
FEP	2	100	100	0.22	[−0.06, 0.49]	0.122	0.00	0.07	0.797
Plasma	4	1,063	1,239	−0.02	[−0.14, 0.10]	0.325	13.50	3.47	0.733
Serum	2	130	130	−1.04	[−3.45, 1.36]	0.395	98.66	74.75	**<0.001**
*Blood VEGF*									
Drug-naïve	1	31	50	−0.77	[−1.23, −0.31]	**0.001**	/	/	/
Medicated	8	786	710	−0.04	[−0.35, 0.27]	0.792	87.70	56.93	**<0.001**
Non-FEP	8	695	684	0.18	[−0.12, 0.48]	0.232	85.60	48.60	**<0.001**
FEP	2	68	64	0.08	[−1.09, 1.25]	0.892	87.90	8.26	**0.004**
Plasma	3	168	169	0.16	[−0.23, 0.55]	0.423	63.21	5.44	**0.066**
Serum	8	713	662	0.08	[−0.28, 0.44]	0.654	90.02	70.11	**<0.001**

CI, confidence interval; FEP, first-episode psychosis; ICAM-1, intercellular adhesion molecule 1; MMP-9, matrix metalloproteinase-9; NGF, nerve growth factor; VCAM-1, vascular cell adhesion molecule 1; VEGF, vascular endothelial growth factor.

a Statistical significance at 0.1 level.

For biomarkers measured in single studies, CSF-to-serum IgG ratio was elevated in the patients (drug-naïve, *p* ≤ 0.002; medicated, *p* ≤ 0.001) (33), while CSF fibrinogen was decreased (*p* = 0.021) (67). CSF IgG index did not differ between groups (33).

### 3.3. Junction proteins and modifiers

Patients with psychosis were found to have higher blood matrix metalloproteinase-9 (MMP-9; SMD, 0.66; 95% CI, 0.46–0.86; *p* < 0.001; Figure 3) (71–79). In subgroup analysis, this pattern remained significant in the plasma (SMD, 0.75; 95% CI, 0.18–1.32; *p* = 0.010) and serum (SMD, 0.65; 95% CI, 0.40–0.90; *p* < 0.001), as well as in patients medicated (SMD, 0.59; 95% CI, 0.16–1.02; *p* = 0.007) and unmedicated (SMD, 1.21; 95% CI, 0.77–1.64; *p* < 0.001). Note that there was only one study with unmedicated patients and two studies with plasma levels in the subgroup analysis. In the one study involving patients post-electroconvulsive therapy (ECT) (79), blood MMP-9 did not differ between groups (SMD, 0.09; 95% CI, −0.53–0.71; *p* = 0.777). Blood zonulin was also elevated in the patients (SMD, 1.17; 95% CI, 0.04–2.30; *p* = 0.043) (80, 81). We found no significant differences between all patients and controls for blood E- (82–84) or P-selectins (82, 84–86), intercellular adhesion molecule 1 (ICAM-1) 83, 86–93), vascular cell adhesion molecule 1 (VCAM-1) (83, 86, 88, 90, 91, 93), tissue inhibitor of matrix metalloproteinase1 (TIMP-1) (79, 94), TIMP-2 (79, 94), or plasminogen activator inhibitor-1 (PAI-1) (95–97). However, blood ICAM-1 was lower in drug-naïve patients (SMD, −0.81; 95% CI, −1.10–-0.53; *p* < 0.001) and patients with FEP (SMD, −0.58; 95% CI, −0.87–-0.28; *p* < 0.001), but elevated in patients with non-FEP (SMD, 0.33; 95% CI, 0.07–0.59; *p* = 0.012). Blood VCAM-1 was significantly reduced in unmedicated patients in one study that was detected as an outlier (Supplementary Figure S2; SMD, −2.27; 95% CI, −2.67–-1.87; *p* < 0.001) (93). Removing this study from the overall analysis still displayed no significant difference in VCAM-1 level between groups (SMD, 0.00; 95% CI, −0.12–0.12; *p* = 0.993), but the heterogeneity was no longer significant.

For biomarkers measured in only one study, CSF MMP-2 (*p* = 0.031) (98) and plasma junctional adhesion molecule A (JAM-A; *p* < 0.001) (86) were reported to be higher in the patients. On the other hand, serum claudin 5 (*p* = 0.037) (81) and MMP-7 (*p* < 0.001) (94) displayed lower levels in patients. No between-group differences were reported for serum VE-cadherin (99), claudin (100), occludin (100), MMP-1 (94), MMP-2 (98), MMP-13 (94); plasma N-cadherin (86); or CSF MMP-3 (67).

### 3.4. Angiogenesis

Blood nerve growth factor (NGF) level did not differ between groups (SMD, −0.77; 95% CI, −1.55–0.01; *p* = 0.054; Figure 4) (44, 48, 101–109). In subgroup analysis, only drug-naïve patients displayed decreased NGF (SMD, −2.16; 95% CI, −4.02–-0.31; *p* = 0.022; Table 2), while no significant differences were detected based on other treatment statuses (i.e., unmedicated or medicated), illness stages (i.e., FEP and non-FEP) or sample origins (i.e., plasma and serum). Blood vascular endothelial growth factor (VEGF) level also did not differ between all patients and controls (SMD, 0.11; 95% CI, −0.17–0.39; *p* = 0.447) (88, 90, 95, 110–117). It was decreased in one study with drug-naïve patients (SMD, −0.77; 95% CI, −1.23–-0.31; *p* = 0.001) (111), but not with other treatment statuses, illness stages, or sample origins.

Decreased NGF in CSF (*p* = 0.038) in patients with psychosis was reported in one study (105).

### 3.5. Neurovascular coupling

No significant differences were detected in CSF neuropeptide Y (NPY; SMD, 0.34; 95% CI, −0.36–1.03; *p* = 0.341; Figure 5) (118, 119) or somatostatin (SST; SMD, −0.19; 95% CI, −1.15–0.77; *p* = 0.700) (120–123) between groups.

However, elevated CSF prostaglandin E (PGE; *p* < 0.001) was reported in one study in the patient group (124).

### 3.6. Heterogeneity

The majority of biomarkers displayed significant heterogeneity between studies, except for CSF albumin, CSF-to-serum albumin ratio, and blood E-selectin and TIMP-1 (Table 1). Among the biomarkers with significant heterogeneity, blood MMP-9, and CSF NPY displayed moderate heterogeneity, while the others all presented high heterogeneity. The possible sources of heterogeneity were investigated in sensitivity, subgroup and meta-regression analyses.

### 3.7. Meta-regression

Conduction of meta-regression analyses was only possible for blood S100B, NGF, and VEGF (Table 3). Analyses revealed positive associations between the effect sizes of blood S100B and age and duration of illness (Supplementary Figure S3). There was no significant correlation between the effect sizes of blood S100B and the percentage of males, the Positive and Negative Syndrome Scale (PANSS) scores, or the doses of medications (Supplementary Table S9). Assessment of the relevance of treatment duration, BMI and smoking status was not possible due to insufficient data, but BMI was positively correlated with the percentage of males (*r* = 0.700, *p* = 0.043) and age (*r* = 0.717, *p* = 0.037). No correlations were found between the effect sizes of blood NGF and age, or between the effect sizes of blood VEGF and the percentage of males or age.

**Table 3:** Meta-regression of molecules examined in ≥10 studies.

Moderator	Number			Meta-regression					
	Studies	Patients	Controls	Slope	95% CI	*p*	Intercept	*Z*	*p*
*Blood S100B*									
Males (%)	26	1,172	967	0.005	[−0.02, 0.03]	0.705	0.763	1.010	0.312
Age	26	1,172	967	0.043	[0.01, 0.07]	**0.008**	−0.519	−0.850	0.394
Illness duration	21	820	709	0.042	[0.00, 0.08]	**0.027**	0.411	1.490	0.135
PANSS, Positive	17	853	739	−0.020	[−0.10, 0.06]	0.623	1.457	1.770	0.077
PANSS, Negative	17	853	739	−0.038	[−0.15, 0.07]	0.493	1.830	1.610	0.107
PANSS, General	15	820	709	−0.060	[−0.12, 0.00]	0.057	3.054	2.800	**0.005**
PANSS, Total	16	775	688	−0.027	[−0.05, 0.00]	0.054	2.967	2.800	**0.005**
CPZeq (mg/day)	10	455	340	−0.002	[−0.01, 0.00]	0.229	2.419	2.220	**0.026**
*Blood NGF*									
Age	10	464	432	−0.014	[−0.09, 0.07]	0.736	−0.141	−0.120	0.907
*Blood VEGF*									
Males (%)	10	862	811	−0.001	[−0.03, 0.03]	0.953	0.125	0.190	0.848
Age	10	862	811	0.010	[−0.02, 0.04]	0.505	−0.483	−0.560	0.579

CI, confidence interval; CPZeq, chlorpromazine equivalent dose; NGF, nerve growth factor; PANSS, Positive and Negative Syndrome Scale; VEGF, vascular endothelial growth factor.

## 4. Discussion

Our study provides the most comprehensive meta-analysis to date investigating the vascular-related biomarkers in psychosis. To our knowledge, this is the first meta-analysis to explore the alterations in blood ACE, TIMP-2, zonulin and PAI-1, as well as CSF ACE, S100B, NPY and SST in individuals with psychotic disorders compared to healthy controls. Overall, we revealed that psychotic disorders are associated with increased CSF-to-serum albumin ratio, and increased blood S100B, MMP-9, and zonulin. While these findings in part support the role of vascular pathology in psychosis, they also raise important questions regarding the implications of various relevant fluid molecules.

A main finding of this study is the alteration of BBB-relevant molecules in psychosis. It has been hypothesized that leakiness of the BBB leads to dysregulated flow of nutrients, metabolites and ions, along with less restricted immunity, which together disrupt the normal cerebral function in psychosis (18). CSF-to-serum albumin ratio remains the gold standard to evaluate BBB permeability. Its increase in the patients, along with the increase in predominantly CNS-originated S100B in blood, points towards the disruption of BBB, allowing molecules to abnormally cross between the circulation and CNS. The consistent elevation of blood S100B across treatment statuses in psychosis suggests the contribution of non-pharmaceutical factors, for example, pathological processes inherent to the disorders. This increase was more evident with greater age and longer disease history, with greater age also associated with higher BMI. Considering the partial release of S100B from adipose tissue (125, 126), further studies are needed to investigate whether more severe disruption of the BBB associates with a longer pathological process itself, greater treatment exposure, and/or weight gain along the course. Though a previous meta-analysis revealed associations between blood S100B level and PANSS scores (127), this was not detected in our analysis with more studies included, despite the low *p* values. It should be noted that evidence from the CSF levels of blood-derived large molecules (i.e., ACE, IgG and fibrinogen) does not consistently support the hypothesis of BBB leakage. Though in fact, fibrinogen, being the largest molecule of the three, displayed abnormal cerebral deposition and degradation in patients with schizophrenia (128). A possible explanation is that for larger molecules, both their leakage from blood and the clearance to CSF are more demanding; thus their fluid levels may be less sensitive to reflect an already compromised barrier. These findings are also limited by the small number of studies.

From a structural perspective, psychosis displayed alterations in tight junctions (TJs) and their modulators in our analyses. It should be noted that although these molecules regulate the sealing of endothelium, they also exist in other tissues, including the gastrointestinal tract. Given the proposed, though still controversial, contributions of dysbiosis and intestinal permeability to the pathophysiology of psychosis (129, 130), caution must be taken when linking these findings to vascular abnormalities. Nevertheless, alterations in these molecules may reflect increased barrier permeability in psychosis. MMPs are a family of endopeptidases upregulated by pro-inflammatory cytokines (131) and are responsible for degrading the extracellular matrix (ECM) and TJs (132). TIMPs, on the other hand, inhibit MMPs and protects the ECM. As a major focus of research among the family, MMP-9 is implicated in endothelial function, immune responses, and synaptic plasticity (132). *MMP-9* gene polymorphism has been associated with schizophrenia (133). The elevation of blood MMP-9 in psychosis in our analyses may in part reflect ongoing inflammation that disrupts endothelial structure. This is in concordance with the alterations in several other TJ modifiers, including zonulin, and MMP-2 and -7, along with the TJ proteins claudin 5 and JAM-A, though the number of studies are small. On the other hand, no alteration was reported for the adherens junction proteins N- or VE-cadherin. Overall, since the investigation of junction proteins in body fluid in psychosis has begun only recently, the evidence is still sparse. It remains unclear to what extent the blood levels of these molecules correlate with their expression on the cell surface, and again where these molecules come from. Therefore, future studies investigating their fluid levels need to address these issues to help complement the existing knowledge of vascular dysfunction in psychosis.

Externally, the interaction between endothelial cells and leukocytes and ECM is partially mediated by cell adhesion molecules (CAMs) and PAI-1 (134). The alterations in blood VCAM-1 and ICAM-1 in patient subgroups likely reflect changes in endothelial functions and inflammation. In contrast, selectins, also family members of the CAMs, did not display alterations. Again the levels of CAMs expression on cell surface is unclear, and the discrepancies in the fluid levels of these molecules prompt further investigation on the demographic and clinical correlates of the participants.

In terms of angiogenesis, the NVU secretes two potent mitogens of the endothelial cells, VEGF and NGF, that modify the BBB (135). NGF has been found to elicit angiogenic responses both directly (136) and indirectly (137) through the upregulation of VEGF and nitric oxide synthase, though it is also involved in other processes including neuronal growth and differentiation (138). VEGF, besides regulating angiogenesis, also promotes the endocytosis of endothelial cells and subsequent BBB disruption (139). Genetic studies in patients with schizophrenia have identified alterations in both molecules (109, 140). While a previous meta-analysis showed robustly decreased blood NGF in schizophrenia patients compared to controls (141), this was only observed in drug-naïve patients in our analysis with stricter inclusion and exclusion criteria, despite including more recent studies. Though CSF NGF was reduced in the patients in one study (105). Similarly, decreased blood VEGF was only detected in one study containing drug-naïve patients (111). Overall, studies examining these molecules are highly heterogenous; addressing the covariates is required to further assess any effects.

Furthermore, the NVU regulates NVC by releasing multiple metabolic by-products, neuropeptides and neuromodulators (142–144). NPY and SST induce vasoconstriction in brain slices (142, 143), whereas PGE and vasoactive intestinal peptide causes vasodilation (144, 145). While the exact mechanisms of actions of these molecules has not been fully understood, it is proposed that they ultimately contribute to neuromodulation (146). Postmortem studies have identified reduced SST expression in the hippocampus and prefrontal cortex of patients with schizophrenia (147, 148). Similarly, NPY expression was reduced in the frontal but not temporal cortex (148, 149), suggestive of regional alterations. However, in our analysis, neither SST nor NPY differed in CSF levels between groups. Only one study revealed increased CSF PGE in the patients (124). One possible explanation is that CSF studies, by nature, lack the ability to assess these molecules acting in a time- and space-restricted manner. SST, in particular, has a very short half-life of 1–3 min (150).

### 4.1. Limitations

Several limitations should be acknowledged in this study. At the study level, the implications of fluid markers are inherently constrained by the lack of both spatial and temporal resolution. While alterations in these molecules prompt further investigation, the lack of changes in fluid levels does not exclude the possibility of local changes at specific time points. From a methodological standpoint, analyses of many of the biomarkers here were hindered by a limited number of studies and insufficient data, potentially resulting in underpowered results. Subgroup and meta-regression analyses thus could not be performed for all covariates. For the majority of biomarkers investigated, there was significant heterogeneity between studies, stemming at least partly from variations in participant demographics, clinical characteristics, and sample origins. Other potential sources of heterogeneity, including the differences in diagnoses across the spectrum, treatment regimens, detection methods and sampling time, could not be thoroughly investigated due to mixed or insufficient data. Notably, while subgroup analyses detected associations between the levels of certain biomarkers and treatment statuses, their associations with individual psychotropic medications, including antipsychotics, could not be further assessed due to the heterogenous treatment regimens and combination therapies administered. Analyses on treatment duration and doses were also extremely limited due to inadequate data. Thus, the observed alterations could be related to treatment effects, especially for biomarkers lacking subgroup analyses on treatment statuses. Moreover, some lifestyle factors known to influence vascular function, such as BMI and smoking, were only assessed in a subset of studies. Additionally, certain studies did not specify the history of substance use or other potentially confounding conditions among participants. The lack of this information made it even more challenging to compare the participants across studies, let alone quantitatively assess their correlations with the fluid markers. Furthermore, the majority of studies had risks of bias, with only 5 studies scoring 8 on NOS, potentially compromising the representativeness of the groups and the comparability between groups.

At the review level, it should be emphasized that although the molecules assessed here are involved in endothelial structure and function, shedding light on the vascular changes in psychosis, some of the blood markers are not vascular-specific. Other possible implications are discussed above. To reduce heterogeneity, we did not include studies containing participants with conditions known to influence vascular functions or controls with family history of psychiatric disorders. However, without a clear understanding of the extent to which these factors influence marker levels, this exclusion strategy may help control confounding variables, or may be overly cautious and reduce the power of the analyses, as in the case of analyses for blood NGF levels.

## 5. Conclusion and perspectives

This meta-analysis of 93 studies and 30 molecules provided evidence of the alterations in vascular-related biomarkers in psychotic disorders. Changes in certain biomarkers were further linked to age, treatment statuses, illness stages and duration, and sample origins. However, implications of the findings were limited by the heterogeneity across studies as well as the versatility and membrane-bound nature of some molecules. Addressing important covariates and confounders would be of extreme importance to clarify if the vascular changes are a primary process of psychosis, secondary to the events along the disease course, or methodologically related. Preferably, future studies should pursue rigorous participant selection and appropriate statistical adjustments to account for the differences in demographic (e.g., BMI and smoking) and clinical characteristics (e.g., illness duration, treatment regimens and symptom severity). Further, to validate the roles of these fluid markers in the vascular pathology in psychosis, future research could adopt a multimodal approach (e.g., by integrating neuroimaging and genetic testing) and incorporate multiple interrelated molecules to explore the relevant pathways and networks. Moreover, there remains a strong need for high-quality longitudinal studies to examine the temporal dynamics of vascular dysfunction in psychosis, and to elucidate the progression and stability of the observed alterations. Longitudinal data could then shed light on the relationships between vascular changes and disease course, treatment response, and clinical outcomes.

## Data availability statement

The original contributions presented in the study are included in the article/Supplementary material, further inquiries can be directed to the corresponding author.

## Author contributions

XL, SH, and PL: concept and design, acquisition, analysis, or interpretation of data, and critical revision of the manuscript for important intellectual content. XL: drafting of the manuscript. XL and SH: statistical analysis. PL: obtained funding and supervision. PL and XL: administrative, technical, or material support. All authors contributed to the article and approved the submitted version.

## Funding

This work was supported by the Tsinghua University Initiative Scientific Research Program (grant number 20161080071). The funder had no role in the design and conduct of the study, or preparation and submission of the manuscript for publication.

## Conflict of interest

The authors declare that the research was conducted in the absence of any commercial or financial relationships that could be construed as a potential conflict of interest.

## Publisher’s note

All claims expressed in this article are solely those of the authors and do not necessarily represent those of their affiliated organizations, or those of the publisher, the editors and the reviewers. Any product that may be evaluated in this article, or claim that may be made by its manufacturer, is not guaranteed or endorsed by the publisher.
