# Supplementary material for: Vascular-related biomarkers in psychosis: a systematic review and meta-analysis
Source: Front Psychiatry. 2023 Aug 25;14:1241422. doi: 10.3389/fpsyt.2023.1241422 (PMC10486913; doi:10.3389/fpsyt.2023.1241422)
Supplement: Supplementary file 1 [file Data_Sheet_1.docx]

[Table S1. PRISMA 2020 checklist 2](#_Toc142172039)

[Table S2. Search term 5](#_Toc142172040)

[Table S3. The adapted version of Newcastle-Ottawa quality assessment Scale (NOS) 6](#_Toc142172041)

[Table S4. Summary of the included studies 7](#_Toc142172042)

[Table S5. Excluded studies and the reasons for exclusion 14](#_Toc142172043)

[Table S6. Summary of the molecules examined in 1 study 19](#_Toc142172044)

[Table S7. Bias assessment according to the adapted version of NOS 20](#_Toc142172045)

[Figure S1. Contour-enhanced funnel plots of molecules examined in ≥10 studies 23](#_Toc142172046)

[Table S8. Egger test results of molecules examined in ≥10 studies 24](#_Toc142172047)

[Figure S2. Galbraith plots of the molecules with outlier studies 25](#_Toc142172048)

[Figure S3. Bubble plots of blood S100B 26](#_Toc142172049)

[Table S9. Treatment regimens of molecules examined in ≥5 studies 27](#_Toc142172050)

[References 31](#_Toc142172051)


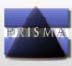
Table S1. PRISMA 2020 checklist

| **Section and Topic** | **Item #** | **Checklist item** | **Location where item is reported** |
| --- | --- | --- | --- |
| **TITLE** | | |  |
| Title | 1 | Identify the report as a systematic review. | p. |
| **ABSTRACT** | | |  |
| Abstract | 2 | See the PRISMA 2020 for Abstracts checklist. | p. |
| **INTRODUCTION** | | |  |
| Rationale | 3 | Describe the rationale for the review in the context of existing knowledge. | p. |
| Objectives | 4 | Provide an explicit statement of the objective(s) or question(s) the review addresses. | p. |
| **METHODS** | | |  |
| Eligibility criteria | 5 | Specify the inclusion and exclusion criteria for the review and how studies were grouped for the syntheses. | p. |
| Information sources | 6 | Specify all databases, registers, websites, organisations, reference lists and other sources searched or consulted to identify studies. Specify the date when each source was last searched or consulted. | p. |
| Search strategy | 7 | Present the full search strategies for all databases, registers and websites, including any filters and limits used. | p. , Table S2 |
| Selection process | 8 | Specify the methods used to decide whether a study met the inclusion criteria of the review, including how many reviewers screened each record and each report retrieved, whether they worked independently, and if applicable, details of automation tools used in the process. | p. |
| Data collection process | 9 | Specify the methods used to collect data from reports, including how many reviewers collected data from each report, whether they worked independently, any processes for obtaining or confirming data from study investigators, and if applicable, details of automation tools used in the process. | p. |
| Data items | 10a | List and define all outcomes for which data were sought. Specify whether all results that were compatible with each outcome domain in each study were sought (e.g. for all measures, time points, analyses), and if not, the methods used to decide which results to collect. | p. |
|  | 10b | List and define all other variables for which data were sought (e.g. participant and intervention characteristics, funding sources). Describe any assumptions made about any missing or unclear information. | p. |
| Study risk of bias assessment | 11 | Specify the methods used to assess risk of bias in the included studies, including details of the tool(s) used, how many reviewers assessed each study and whether they worked independently, and if applicable, details of automation tools used in the process. | p. |
| Effect measures | 12 | Specify for each outcome the effect measure(s) (e.g. risk ratio, mean difference) used in the synthesis or presentation of results. | p. |
| Synthesis methods | 13a | Describe the processes used to decide which studies were eligible for each synthesis (e.g. tabulating the study intervention characteristics and comparing against the planned groups for each synthesis (item #5)). | p. |
|  | 13b | Describe any methods required to prepare the data for presentation or synthesis, such as handling of missing summary statistics, or data conversions. | p. |
|  | 13c | Describe any methods used to tabulate or visually display results of individual studies and syntheses. | p. |
|  | 13d | Describe any methods used to synthesize results and provide a rationale for the choice(s). If meta-analysis was performed, describe the model(s), method(s) to identify the presence and extent of statistical heterogeneity, and software package(s) used. | p. |
|  | 13e | Describe any methods used to explore possible causes of heterogeneity among study results (e.g. subgroup analysis, meta-regression). | p. |
|  | 13f | Describe any sensitivity analyses conducted to assess robustness of the synthesized results. | p. |
| Reporting bias assessment | 14 | Describe any methods used to assess risk of bias due to missing results in a synthesis (arising from reporting biases). | p. |
| Certainty assessment | 15 | Describe any methods used to assess certainty (or confidence) in the body of evidence for an outcome. | p. |
| **RESULTS** | | |  |
| Study selection | 16a | Describe the results of the search and selection process, from the number of records identified in the search to the number of studies included in the review, ideally using a flow diagram. | p., Figure 1 |
|  | 16b | Cite studies that might appear to meet the inclusion criteria, but which were excluded, and explain why they were excluded. | Table S5 |
| Study characteristics | 17 | Cite each included study and present its characteristics. | Table S4 |
| Risk of bias in studies | 18 | Present assessments of risk of bias for each included study. | Table S7 |
| Results of individual studies | 19 | For all outcomes, present, for each study: (a) summary statistics for each group (where appropriate) and (b) an effect estimate and its precision (e.g. confidence/credible interval), ideally using structured tables or plots. | Figures 2-5 |
| Results of syntheses | 20a | For each synthesis, briefly summarise the characteristics and risk of bias among contributing studies. | p. |
|  | 20b | Present results of all statistical syntheses conducted. If meta-analysis was done, present for each the summary estimate and its precision (e.g. confidence/credible interval) and measures of statistical heterogeneity. If comparing groups, describe the direction of the effect. | Figures 2-3 & S1 |
|  | 20c | Present results of all investigations of possible causes of heterogeneity among study results. | p. |
|  | 20d | Present results of all sensitivity analyses conducted to assess the robustness of the synthesized results. | p. |
| Reporting biases | 21 | Present assessments of risk of bias due to missing results (arising from reporting biases) for each synthesis assessed. | Figure S1, Table S8 |
| Certainty of evidence | 22 | Present assessments of certainty (or confidence) in the body of evidence for each outcome assessed. | Figures 2-5 |
| **DISCUSSION** | | |  |
| Discussion | 23a | Provide a general interpretation of the results in the context of other evidence. | p. |
|  | 23b | Discuss any limitations of the evidence included in the review. | p. |
|  | 23c | Discuss any limitations of the review processes used. | p. |
|  | 23d | Discuss implications of the results for practice, policy, and future research. | p. |
| **OTHER INFORMATION** | | |  |
| Registration and protocol | 24a | Provide registration information for the review, including register name and registration number, or state that the review was not registered. | p. |
|  | 24b | Indicate where the review protocol can be accessed, or state that a protocol was not prepared. | p. |
|  | 24c | Describe and explain any amendments to information provided at registration or in the protocol. | – |
| Support | 25 | Describe sources of financial or non-financial support for the review, and the role of the funders or sponsors in the review. | p. |
| Competing interests | 26 | Declare any competing interests of review authors. | p. |
| Availability of data, code and other materials | 27 | Report which of the following are publicly available and where they can be found: template data collection forms; data extracted from included studies; data used for all analyses; analytic code; any other materials used in the review. | Figures 2-5, Supplement |

*From:*  Page MJ, McKenzie JE, Bossuyt PM, Boutron I, Hoffmann TC, Mulrow CD, et al. The PRISMA 2020 statement: an updated guideline for reporting systematic reviews. BMJ 2021;372:n71. doi: 10.1136/bmj.n71

For more information, visit: <http://www.prisma-statement.org/>

Table S2. Search term

The following search term including titles, abstracts, keywords and medical subject headings (MeSH) was used:

(psychosis OR psychotic OR schizophren* OR schizoaffective OR delusion* OR UHR OR ARMS OR ultra high risk OR clinical high risk OR genetic high risk OR prodrom*)

AND

(cerebrospinal fluid OR CSF OR blood OR serum OR plasma OR peripheral OR circulat* OR fluid OR molecular OR biochemical)

AND

(neurovascul* OR cerebrovascul* OR blood-brain barrier OR vascul* OR endotheli* OR albumin OR QAlb OR immunoglobulin G OR IgG OR fibrinogen OR angiotensin-converting enzyme OR angiogenin OR S100B OR plasminogen activator inhibitor-1 OR PAI-1 OR matrix metalloproteinase* OR MMP OR tissue inhibitor of metalloproteinase* OR TIMP OR cell adhesion molecule* OR ICAM OR VCAM OR selectin OR cadherin OR claudin OR zonulin OR glycocalyx OR syndecan OR heparan sulfate OR chondroitin sulfate OR hyaluronan OR p-glycoprotein OR ATP Binding Cassette OR ABCB1 OR AQP4 OR growth factor OR VEGF OR NGF OR heart-type fatty acid-binding protein OR hFABP OR vasoactive intestinal peptide OR neuropeptide Y OR somatostatin OR nitric oxide OR prostaglandin OR PGE)

Table S3. The adapted version of Newcastle-Ottawa quality assessment Scale (NOS)

| **Rating item** | **Definition** |
| --- | --- |
| **Selection** |  |
| 1. Adequate case definition | 1 star is awarded if the diagnosis was independently validated by ≥2 investigators according to a recognized diagnostic tool |
| 2. Representativeness of cases | 1 star is awarded if all cases were included over a defined period of time, or in a defined catchment area or healthcare organization |
| 3. Selection of controls | 1 star is awarded if controls included were in the same community as cases and would be cases if had outcome |
| 4. Definition of controls | 1 star is awarded if controls have no current or past history of psychiatric illness |
| **Comparability** |  |
| 1. Study controls for age | 1 star is awarded if the ages of controls and patients were matched and/or adjusted for in the analysis |
| 2. Study controls for other factors | 1 star awarded if other factors (e.g., sex) of controls and patients were matched and/or adjusted for in the analysis |
| **Exposure** |  |
| 1. Ascertainment of exposure | 1 star is awarded if investigators responsible for biomarker measurement were blinded to the case-control status |
| 2. Same method of ascertainment for cases and controls | 1 star is awarded if the same methods of sample collection, handling, and measurement were used for both patients and controls |

Table S4. Summary of the included studies

| **Study** | **Patients** | | | | | |  | **Controls** | | |  | **Measurement** | |
| --- | --- | --- | --- | --- | --- | --- | --- | --- | --- | --- | --- | --- | --- |
|  | **N** | **Diagnosis (tool)** | **Males (%)** | **Age** | **Illness duration** | **Treatment** |  | **N** | **Males (%)** | **Age** |  | **Molecule** | **Method** |
| Ajami, 2014 (1) | 26 | SZ (DSM-IV) | NA | 9.49 ± 33.61 | NA | UM (BL) → M |  | 26 | NA | 8.86 ± 33.92 |  | Serum NGF | ELISA |
| Ali, 2017 (2) | 44 | SZ (DSM-IV) | 77.27 | 25.10 ± 4.00 | 2.00 ± 0.46 | UM |  | 50 | 64.00 | 26.10 ± 3.90 |  | Serum MMP-9 | ELISA |
| Arabska, 2019 (3) | 64 | SZ (ICD-10) | 45.31 | 49.00 ± 8.19 | NA | 17 M for 15.20 ± 8.70 years; others NA |  | 32 | 53.13 | 51.00 ± 8.90 |  | Serum MMP-9 | ELISA |
| Balõtšev, 2017 (4) | 105 | SZ (ICD-10) | 42.86 | 53.10 ± 10.90 | 19.90 ± 9.80 | M for ≥1 month |  | 148 | 45.95 | 51.30 ± 7.57 |  | Serum VEGF | CLIA |
| Bersani, 2004 (5) | 10 | SZ (DSM-IV) | 100.00 | 24.72 ± 2.59 | NA | UM for 15 days |  | 23 | 60.87 | 25.00 ± 2.36 |  | Plasma NGF | ELISA |
| Bissette, 1986 (6) | 10 | SZ (DSM-III) | 30.00 | 46.60 ± 11.70 | NA | UM for ≥2 weeks |  | 10 | 50.00 | 34.20 ± 10.10 |  | CSF SST | RIA |
| Bocchio-Chiavetto, 2018(7) | 53 | FEP (ICD-10) | 64.15 | 28.70 ± 8.60 | NA | M, UM |  | 49 | 51.02 | 32.00 ± 5.10 |  | Serum PAI-1, VEGF | xMAP |
| Cai, 2020 (8) | 78 | 51 SZ, 27 SZA (DSM-IV-TR) | 61.54 | 35.30 ± 8.70 | 12.30 ± 7.17 | M for ≥1 year |  | 73 | 47.95 | 32.20 ± 8.51 |  | Plasma ICAM-1 | xMAP |
| Chen, 2017a (9) | 62 | SZ (DSM-IV) | 38.71 | 35.31 ± 11.52 | NA | 7 DN; 27 M for <2 weeks → UM for ≥1month; 28 UM for ≥1month |  | 40 | 50.00 | 25.68 ± 6.55 |  | Serum S100B | ELISA |
| Chen, 2017b (10) | 34 | SZ (DSM-IV) | 44.12 | 34.35 ± 10.89 | 6.06 ± 6.16 | UM for ≥2 weeks |  | 30 | 53.33 | 35.60 ± 12.90 |  | Serum PAI-1 | ELISA |
| Chenniappan, 2022 (11) | 62 | SSD (DSM-5) | 50.00 | 32.21 ± 8.86 | 2 (0.08–20) | 37 DN, 25 UM for ≥6 weeks (BL) → M for 6 weeks |  | 62 | 50.00 | 38.70 ± 8.00 |  | Serum MMP-9 | ELISA |
| Dai, 2020 (12) | 83 | FEP (DSM-IV) | 54.22 | 23.65 ± 6.70 | 1.04 ± 1.25 | DN |  | 60 | 53.33 | 24.60 ± 3.80 |  | Serum NGF, S100B | ELISA |
| Devanarayanan, 2016 (13) | 40 | SZ (DSM-IV-TR) | 100.00 | 28.30 ± 6.70 | 3.60 ± 3.70 | 24 DN, 16 UM for ≥4 weeks |  | 40 | 100.00 | 26.60 ± 4.50 |  | Serum MMP-9 | ELISA |
| Elmi, 2019 (14) | 30 | 9 SZ, 21 SZA (DSM-5) | NA | NA | NA | M |  | 20 | 35.00 | 39.10 ± 2.51 |  | Plasma PAI-1 | ELISA |
| Falcone, 2015 (15) | 67 | PNOS, SZPH, SZ (DSM-IV-TR) | NA | NA | NA | NA |  | 22 | 45.45 | 16.59 ± 1.01 |  | Serum S100B | ELISA |
| Gattaz, 2000 (16) | 23 | SZ (DSM-IV) | 69.57 | 36.00 ± 9.00 | 17.00 ± 7.00 | M |  | 23 | 69.57 | 44.00 ± 17.00 |  | Plasma S100B | ILMA |
| Gerasimou, 2018 (17) | 13 | FEP (DSM-IV) | 61.54 | 23.70 ± 4.00 | 0.67 ± 0.42 | DN (BL) → M for 6 weeks |  | 10 | 50.00 | 65.60 ± 3.90 |  | Serum S100B | ELISA |
| Gerner, 1985 (18) | 72 | SZ (RDC) | NA | NA | NA | UM for ≥2 weeks |  | 31 | NA | NA |  | CSF SST | RIA |
| Goff, 2018 (19) | 69 | FEP (DSM-IV-TR) | NA | NA | NA | 67 DN, 4 M (BL) → M for 8 weeks |  | 61 | NA | NA |  | Plasma S100B | CA |
| Gokulakrishnan, 2022(20) | 40 | SZ (DSM-5) | 47.50 | 33.30 ± 8.10 | NA | 15 DN, 25 M |  | 20 | 55.00 | 32.50 ± 6.80 |  | Plasma Zonulin | ELISA |
| Hayes, 2014 (21) | 46 | SZ (DSM-IV) | 78.26 | 25.80 ± 5.43 | NA | DN |  | 35 | 60.00 | 26.40 ± 3.55 |  | CSF ACE, fibrinogen, MMP-3 | xMAP |
| Hendouei, 2016 (22) | 19 | SZ (DSM-IV-TR, ICD-10) | 68.42 | 34.05 ± 9.90 | 9.40 ± 9.41 | M for ≥6 months (BL) → M for 6 weeks |  | 25 | 72.00 | 34.20 ± 8.30 |  | Serum S100B | ELISA |
| Hidese, 2020 (23) | 94 | SZ (DSM-IV) | 59.57 | 40.50 ± 10.10 | 15.70 ± 9.60 | 83 M, 11 UM |  | 118 | 55.93 | 42.40 ± 15.30 |  | CSF S100B | xMAP |
| Hong, 2016 (24) | 41 | SZ (ICD-10) | 43.90 | 37.00 ± 11.30 | 11.60 ± 8.90 | UM for ≥1 month |  | 33 | 30.30 | 35.15 ± 13.72 |  | Plasma S100B | ELISA |
| Huang, 2022 (25) | 78 | SZ (ICD-10) | 57.69 | 24.73 ± 5.10 | 0.83 (0.67–1.08) | UM |  | 71 | 46.48 | 26.30 ± 5.10 |  | Serum NGF, S100B | ELISA |
| Iwata, 2007 (26) | 23 | SZ (DSM-IV) | 56.52 | 32.70 ± 10.40 | 4.10 ± 4.70 | DN or UM for ≥6 months |  | 36 | 63.89 | 35.80 ± 10.90 |  | Serum E-, P-selectin | ELISA |
| Jockers-Scherubl, 2003 (27) | 76 | SZ (DSM-IV) | 52.63 | 33.35 | NA | DN |  | 61 | 57.38 | 31.49 ± 8.63 |  | Serum NGF | ELISA |
| Jockers-Scherubl, 2006 (28) | 66 | SZ (DSM-IV) | 65.15 | 36.00 ± 9.40 | NA | DN (BL) → M for ≥4 weeks |  | 51 | 47.06 | 29.40 ± 8.30 |  | Serum NGF | ELISA |
| Kale, 2009 (29) | 30 | FEP (DSM-IV) | 46.67 | 33.00 ± 2.08 | NA | DN |  | 42 | 42.86 | 32.83 ± 2.05 |  | Plasma NGF | ELISA |
|  | 28 | FEP (DSM-IV) | NA | NA | NA | DN |  | 14 | NA | NA |  | CSF NGF | ELISA |
| Kannan, 2017 (30) | 175 | SZ, SZPH, SZA (DSM-IV) | NA | NA | NA | M |  | 91 | NA | NA |  | Serum S100B | xMAP |
| Kavzoglu, 2013 (31) | 50 | FEP (DSM-IVTR) | 50.00 | 30.14 ± 7.50 | NA | DN (BL) → M for 3 months |  | 50 | 42.00 | 30.90 ± 6.42 |  | Plasma E-selectin, ICAM-1, VCAM-1 | ELISA |
| Keshri, 2021 (32) | 124 | SZ (DSM-5) | 45.16 | 36.18 ± 9.73 | 3 (0–20) | 50 DN, 74 UM |  | 124 | 43.55 | 37.62 ± 8.23 |  | Serum MMP-9 | ELISA |
| Klaus, 2021 (33) | 19 | SZ (DSM-IV-TR) | NA | NA | NA | NA |  | 20 | 40.00 | 39.95 ± 9.65 |  | Plasma ICAM-1, VCAM-1, VEGF | ECLIA |
| Kozłowska, 2021 (34) | 58 | SZ (DSM-IV, ICD-10) | NA | NA | NA | M |  | 29 | 65.52 | 37.60 ± 10.60 |  | Serum S100B | ELISA |
| Krönig, 2005 (35) | 70 | 66 SZ, 1 SZPH, 3 SZA (DSM-IV) | 58.57 | 31.17 ± 10.62 | NA | UM for >8 weeks |  | 128 | 54.69 | 30.05 ± 9.70 |  | Serum ICAM-1 | ELISA |
| Kudo, 2019 (36) | 249 | SZ (DSM-IV) | 50.60 | 42.50 ± 14.00 | 12.30 ± 10.80 | DN, UM, M |  | 257 | 50.58 | 41.80 ± 12.70 |  | Plasma MMP-9 | ELISA |
| Lara, 2001 (37) | 20 | SZ (DSM-IV) | 65.00 | 31.00 ± 8.00 | 9.50 ± 7.60 | 1 DN, 19 UM for ≥1 week |  | 20 | 65.00 | 31.00 ± 9.00 |  | Serum S100B | ILMA |
| Lee, 2009 (38) | 36 | SZ (DSM-IV) | 41.67 | 31.30 ± 7.80 | 4.72 ± 4.93 | 24 DN, 12 UM for ≥4 weeks (BL) → M for 6 weeks |  | 36 | 41.67 | 31.30 ± 7.90 |  | Plasma NGF | ELISA |
| Lee, 2015 (39) | 50 | SZ (DSM-IV) | 44.00 | 33.10 ± 8.90 | NA | DN, UM (BL) → M for 6 weeks |  | 50 | 48.00 | 32.70 ± 7.20 |  | Plasma VEGF | ELISA |
| Lin, 2018 (40) | 23 | SZ (ICD-10) | 78.26 | 30.17 ± 10.89 | 0–12 | 18 DN, 5 UM for ≥6 months |  | 52 | 63.46 | 32.15 ± 10.54 |  | Serum VEGF | xMAP |
| Ling, 2007 (41) | 57 | SZ (DSM-IV, ICD-10) | 47.37 | 33.50 ± 11.35 | 8.00 ± 9.01 | UM for ≥2 weeks (BL) → M for 12 weeks |  | 60 | 50.00 | 34.92 ± 6.68 |  | Plasma S100B | ELISA |
| Liu, 2020 (42) | 165 | SZ (DSM-5) | 53.94 | 42.30 ± 11.49 | 18.38 ± 11.44 | M |  | 72 | 52.78 | 40.60 ± 8.30 |  | Serum VEGF | ELISA |
| Liu, 2021 (43) | 138 | SZ (DSM-IV) | 52.17 | 45.09 ± 12.47 | 21.74 ± 13.51 | M for ≥3 months |  | 160 | 33.75 | 41.43 ± 9.50 |  | Serum VEGF | ELISA |
| Masopust, 2013 (44) | 36 | 21 SZ, 1 DD, 14 ASP (ICD-10) | 52.78 | 28.10 ± 8.00 | NA | DN (BL) → M for 3 months → M for 1 year |  | 37 | 51.35 | 28.10 ± 8.30 |  | Plasma P-selectin | ELISA |
| Mathe, 1980 (45) | 8 | SZ (RDC) | 100.00 | 26.70 ± 7.07 | NA | UM for 2 weeks |  | 9 | 100.00 | 31.40 ± 14.10 |  | CSF PGE | RIA |
| Milleit, 2016 (46) | 17 | 11 FEP, 6 SZ (DSM-IV) | 35.29 | 34.58 ± 10.99 | NA | 10 DN, 7 UM for ≥4 days |  | 22 | 40.91 | 34.70 ± 10.30 |  | Serum S100B | ILMA |
| Mohite, 2017 (47) | 39 | SZ (DSM-5) | 74.36 | 32.79 ± 12.05 | NA | M |  | 19 | 31.58 | 33.68 ± 9.90 |  | Plasma E-, P-selectin | ELISA |
| Morera-Fumero, 2017 (48) | 65 | SZ (DSM-IV) | 55.38 | 36.70 ± 10.20 | 12.30 ± 10.00 | M (BL) → M (discharge) |  | 65 | 70.77 | 39.60 ± 9.80 |  | Serum S100B | ELISA |
| Morera-Fumero, 2022 (49) | 23 | SZ (DSM-IV) | 69.57 | 36.80 ± 9.20 | 12.20 ± 10.30 | M |  | 23 | 78.26 | 39.00 ± 9.50 |  | Serum S100B | ELISA |
| Murphy, 2014 (50) | 15 | FEP (DSM-IV) | 66.67 | 18.60 ± 3.30 | NA | 8 DN, 7 M for ≤1 week (BL) → M for 8 weeks |  | 15 | NA | 18.50 ± 3.10 |  | Serum VEGF | xMAP |
| Neugebauer, 2019 (51) | 18 | SZ (ICD-10) | 61.11 | 36.94 ± 9.90 | 12.58 ± 9.67 | M |  | 19 | 63.16 | 35.79 ± 11.56 |  | Serum NGF | ELISA |
| Nguyen, 2018 (52) | 99 | 55 SZ, 44 SZA (DSM-IV-TR) | 49.49 | 48.70 ± 10.10 | 25.60 ± 11.10 | M |  | 99 | 49.49 | 47.70 ± 12.00 |  | Plasma ICAM-1, VCAM-1, VEGF | ECL |
| Niitsu, 2014 (53) | 63 | SZ (DSM-IV) | 41.27 | 35.90 ± 8.20 | 9.10 ± 7.30 | M for ≥8 weeks |  | 52 | 48.08 | 34.90 ± 7.30 |  | Serum MMP-9 | ELISA |
| O'Connell, 2013 (54) | 97 | SZ (DSM-IV) | 70.10 | 42.50 ± 12.20 | NA | M |  | 27 | 37.04 | 42.40 ± 10.30 |  | Serum S100B | ELISA |
| Omori, 2020 (55) | 86 | SZ (DSM-IV) | 60.47 | 40.70 ± 10.40 | NA | M |  | 106 | 54.72 | 42.60 ± 15.40 |  | CSF MMP-2, -7, -8, -10 | xMAP |
| Peters, 1990 (56) | 35 | SZ (DSM-III) | 100.00 | 33.90 ± 7.80 | 10.50 ± 6.83 | 4 DN, 31 UM (M [BL] → UM) |  | 31 | 61.29 | 28.50 ± 7.20 |  | CSF NPY | RIA |
| Pillai, 2017 (57) | 96 | SZ (DSM-IV) | 61.46 | 35.70 ± 8.40 | 12.90 ± 7.60 | M for ≥1 year |  | 83 | 51.81 | 31.90 ± 8.50 |  | Serum VEGF | ELISA |
| Qi, 2009 (58) | 63 | SZ (DSM-IV) | 68.25 | 50.80 ± 6.80 | 25.40 ± 7.20 | M for ≥12 months |  | 50 | 58.00 | 49.60 ± 5.50 |  | Serum S100B | ELISA |
| Qing, 2021 (59) | 47 | FEP (DSM-IVR) | NA | NA | NA | DN |  | 50 | NA | NA |  | Plasma S100B | CA |
| Radu, 2020 (60) | 50 | FEP (DSM-5) | 46.00 | 29.70 ± 6.60 | NA | DN (BL) → M for 6 months |  | 50 | 48.00 | 53.22 ± 13.07 |  | Serum ICAM-1, VCAM-1 | ELISA |
| Reinikainen, 1990 (61) | 11 | SZ (DSM-III) | 9.09 | 59.00 ± 4.00 | NA | M for ≥2 weeks |  | 8 | 37.50 | 65.00 ± 4.00 |  | CSF SST | RIA |
| Roos, 1985 (62) | 32 | SZ (RDC) | NA | NA | NA | UM for ≥2 weeks |  | 31 | NA | NA |  | CSF albumin, IgG | EID |
| Rothermundt, 2001 (63) | 26 | SZ (DSM-IV) | 38.46 | 37.00 ± 12.90 | 9.96 ± 10.35 | UM for ≥6 months (BL) → M for 6 weeks |  | 26 | 38.46 | 37.00 ± 12.90 |  | Plasma S100B | IFMA |
| Rothermundt, 2004 (64) | 98 | SZ (ICD-10) | 57.14 | 42.10 ± 11.10 | >2 | UM for 1 week (BL) → M for 12 weeks → M for 24 weeks |  | 98 | 57.14 | 42.10 ± 11.10 |  | Serum S100B | ILMA |
| Rothermundt, 2007 (65) | 12 | SZ (DSM-IV) | 91.67 | 25.33 ± 4.75 | 1.93 ± 1.36 | 9 DN, 3 M |  | 12 | 91.67 | 25.33 ± 4.75 |  | Serum S100B | ILMA |
| Rubinow, 1986 (66) | 44 | SZ (DSM-III) | NA | 24.10 | NA | UM for ≥2 weeks |  | 47 | NA | NA |  | CSF SST | RIA |
| Sarandol, 2007 (67) | 40 | SZ (DSM-IV) | 45.00 | 34.90 ± 9.90 | 6.70 ± 6.40 | 9 {Hayes, 2014 #91525}{Omori, 2020 #92968}DN, 31 UM for ≥3 weeks (BL) → M for 6 weeks |  | 35 | 48.57 | 33.50 ± 9.20 |  | Serum S100B | ELISA |
| Schmitt, 2005 (68) | 41 | SZ (DSM-IV) | 58.54 | 63.30 ± 7.00 | 35.30 ± 11.40 | M |  | 23 | 65.22 | 64.50 ± 9.80 |  | Serum S100B | ILMA |
| Schroeter, 2003 (69) | 30 | SZ (DSM-IV, ICD-10) | 46.67 | 34.80 ± 12.40 | 8.90 ± 8.80 | 14 UM, 16 M |  | 15 | 53.33 | 34.20 ± 5.60 |  | Serum S100B | ILMA |
| Schroeter, 2009 (70) | 20 | SZ (DSM-IV, ICD-10) | 45.00 | 34.60 ± 12.70 | 8.40 ± 9.60 | 5 UM, 15 M (BL) → M (discharge) |  | 19 | 52.63 | 37.90 ± 10.20 |  | Serum S100B | ILMA |
| Schwarz, 1998 (71) | 24 | 13 FEP, 17 SZ (DSM-III, ICD-10) | NA | NA | NA | 13 DN, 17 UM for ≥4 months (BL) → M for 0.5–7 months |  | 31 | 54.84 | 28.60 ± 6.47 |  | Serum ICAM-1 | ELISA |
| Seitz-Holland, 2022 (72) | 30 | SZ (DSM-5) | 63.33 | 33.07 ± 9.48 | 7.96 ± 6.45 | M |  | 34 | 61.76 | 32.50 ± 9.10 |  | Plasma MMP-9 | ELISA |
| Severance, 2015 (73) | 54 | FEP (DSM-IV) | NA | NA | NA | DN, M |  | 60 | NA | NA |  | CSF albumin, IgG; CSF:serum albumin, IgG; CSF IgG index | NA |
| Sheikh, 2023 (74) | 895 | SSD (DSM-IV) | NA | NA | NA | 525 M, others NA |  | 1070 | NA | NA |  | Plasma ICAM-1, JAM-A, NCAD, P-selectin, VCAM-1 | ELISA |
| Shibasaki, 2016 (75) | 13 | 10 SZ, 3 SZA (DSM-IV-TR) | 46.15 | 46.90 ± 15.10 | 15.50 ± 12.10 | M (BL) → ECT |  | 40 | 35.00 | 54.20 ± 13.90 |  | Serum MMP-2, -9; TIMP-1, -2 | ELISA |
| Smirnova, 2019 (76) | 33 | SZ (ICD-10) | 33.33 | 34.69 ± 8.58 | 10.37 ± 8.43 | UM for ≥6 months |  | 24 | 25.00 | 28 (21–55) |  | Serum Cadherin 5 | ELISA |
| Stefanovic, 2016 (77) | 80 | SZ (DSM-IV) | 42.50 | 32.75 ± 11.03 | 9.25 ± 8.37 | UM for ≥4 weeks |  | 80 | 41.25 | 33.21 ± 7.86 |  | Serum ICAM-1, VCAM-1 | ELISA |
| Steiner, 2006 (78) | 12 | FEP (DSM-IV, ICD-10) | 58.33 | 24.00 ± 7.00 | 0.40 ± 0.21 | M |  | 17 | 52.94 | 25.00 ± 8.00 |  | CSF & serum S100B; CSF:serum albumin | ILMA |
| Steiner, 2009 (79) | 26 | SZ (DSM-IV) | 65.38 | 34.70 ± 11.30 | 8.00 ± 9.00 | 11 UM for ≥6 weeks, 15 M for 26 ± 21 days (BL) → M for 6 weeks |  | 32 | 62.50 | 34.40 ± 10.80 |  | Serum S100B | ILMA |
| Turkmen, 2021 (80) | 41 | SZ (DSM-5) | 100.00 | 37.47 ± 10.72 | NA | M for 1 day (BL) → M for 20 days |  | 43 | 100.00 | 36.27 ± 10.14 |  | Serum NGF | ELISA |
| Tylec, 2021 (81) | 25 | SZ (DSM-5, ICD-10) | 48.00 | 61.60 ± 8.90 | 32.60 ± 6.20 | M |  | 25 | 44.00 | 65.30 ± 4.80 |  | Serum MMP-1, -7, -13; TIMP-1, -2 | ELISA |
| Usta, 2021 (82) | 50 | SZ (DSM-5) | 74.00 | 38.30 ± 10.70 | 14.20 ± 9.60 | M |  | 50 | 62.00 | 40.10 ± 8.50 |  | Serum claudin 5, zonulin | ELISA |
| Uzbay, 2013 (83) | 18 | SZ (DSM-IV) | 61.11 | 37.39 ± 12.60 | 9.83 ± 10.06 | UM for ≥6 months |  | 19 | 57.89 | 33.89 ± 6.19 |  | Plasma S100B | ELISA |
| Wahlbeck, 1993(84) | 14 | SZ (DSM-IIIR) | 64.29 | 42.80 ± 11.00 | 18.50 ± 12.20 | M |  | 9 | 55.56 | 38.30 ± 7.80 |  | CSF & serum ACE | IBA |
| Wahlbeck, 1997 (85) | 9 | 5 SZ, 3 SZPH, 1 SZA (DSM-IV) | 44.44 | 34.30 ± 11.40 | NA | 1 UM for ≥3 months, 8 M (BL) → M |  | 9 | 44.44 | 34.60 ± 11.70 |  | CSF & serum ACE | IBA |
| Wahlbeck, 1998 (86) | 43 | SSD (DSM-IV) | NA | 33.92 ± 9.57 | NA | 7 UM, 36 M |  | 19 | 57.89 | 32.40 ± 10.60 |  | CSF & serum ACE | IBA |
| Widerlov, 1988 (87) | 38 | SZ (RDC) | NA | NA | NA | 21 DN, 14 UM for >2 weeks → 16 M for ≥1 month |  | 20 | 45.00 | NA |  | CSF NPY | RIA |
| Wiesmann, 1999 (88) | 20 | SZ (DSM-IV, ICD-10) | 40.00 | 35.70 ± 10.70 | 8.30 ± 5.10 | M |  | 20 | 40.00 | 35.70 ± 10.70 |  | Plasma S100B | IFMA |
| Xiao, 2018 (89) | 118 | 47 FEP, 71 SZ (DSM-5, ICD-10) | 52.54 | 34.20 ± 11.90 | 8.80 ± 9.10 | 47 DN, 71 UM for ≥4 weeks → M for 6 weeks |  | 83 | 56.63 | 34.70 ± 10.90 |  | Serum VEGF | ELISA |
| Yazla, 2022 (90) | 75 | SZ (DSM-5) | 58.67 | 45.07 ± 8.56 | ≥1 | M for ≥6 months |  | 41 | 53.66 | 43.90 ± 7.62 |  | Serum claudin, occludin | ELISA |
| Zakharyan, 2014 (91) | 145 | 25 FEP, 120 SZ (DSM-IV-TR, ICD-10) | NA | NA | NA | 25 DN, 120 M |  | 120 | NA | NA |  | Plasma NGF | ELISA |
| Zhang, 2010a (92) | 50 | SZ (DSM-IV) | 72.00 | 50.50 ± 7.00 | 26.60 ± 8.70 | M for ≥12 months |  | 60 | 68.33 | 49.40 ± 9.90 |  | Serum S100B | ELISA |
| Zhang, 2010b (93) | 162 | SZ (DSM-IV) | 61.11 | 40.13 ± 13.76 | 14.50 ± 13.81 | 80 DN, 82 M for ≥12 months |  | 97 | 60.82 | 37.90 ± 9.00 |  | Serum S100B | ELISA |

Table shows mean ± standard deviation or median (interquartile range). ASP, acute schizophreniform psychosis. BL, baseline. CA, cytokine array. CLIA, chemiluminescent immunoassay. DD, delusional disorder. DN, drug-naïve. EID, electroimmunodiffusion. ELISA, enzyme-linked immunosorbent assay. FEP, first-episode psychosis. IBA, inhibitor binding assay. IFMA, immunofluorometric assay. ILMA, immunoluminometric assay. M, medicated. NA, not available. PNOS, psychotic disorder not otherwise specified. RIA, radioimmunoassay. SSD, schizophrenia spectrum disorder. SZ, schizophrenia. SZA, schizoaffective disorder. SZPH, schizophreniform disorder. UM, unmedicated.

Table S5. Excluded studies and the reasons for exclusion

| **Study** | **Reason(s) for exclusion** |
| --- | --- |
| Ak, 2013 | Conference abstract |
| Arranz, 2016 | Conference abstract |
| Chiavetto, 2016 | Conference abstract |
| Chow, 2014 | Conference abstract |
| Cotter, 2015 | Conference abstract |
| Diaz-Mesa, 2017 | Conference abstract |
| Dickerson, 2015 | Conference abstract |
| Dinh, 2015 | Conference abstract |
| Djordjevic, 2014 | Conference abstract |
| Dorofeikova, 2016 | Conference abstract |
| Dorofeykov, 2015 | Conference abstract |
| Dorofeykov, 2017 | Conference abstract |
| Eyler, 2016 | Conference abstract |
| Falcone, 2010 | Conference abstract |
| Falcone, 2011 | Conference abstract |
| Falcone, 2015b | Conference abstract |
| Fekkes, 2009 | Conference abstract |
| Gardner, 2014 | Conference abstract |
| Goff, 2017 | Conference abstract |
| Goldwaser, 2021 | Conference abstract |
| Goncalves, 2011 | Conference abstract |
| Hattori, 2016 | Conference abstract |
| Hohmann, 2014 | Conference abstract |
| Jeffries, 2017 | Conference abstract |
| Kalayci, 2014 | Conference abstract |
| Kavzoglu, 2011 | Conference abstract |
| Kelly, 2017 | Conference abstract |
| Khan, 1995 | Conference abstract |
| Kim, 2006 | Conference abstract |
| Kim, 2008 | Conference abstract |
| Kim, 2012 | Conference abstract |
| Kim, 2015 | Conference abstract |
| Kroken, 2017 | Conference abstract |
| Lizano, 2015 | Conference abstract |
| Lu, 2013 | Conference abstract |
| Maly, 2011 | Conference abstract |
| Maly, 2012 | Conference abstract |
| Maly, 2013 | Conference abstract |
| Masopust, 2010 | Conference abstract |
| Masopust, 2011 | Conference abstract |
| Mednova, 2021 | Conference abstract |
| Miller, 2017 | Conference abstract |
| Morera-Fumero, 2010 | Conference abstract |
| Morera-Fumero, 2016 | Conference abstract |
| O'Connell, 2012 | Conference abstract |
| Oliveira, 2016 | Conference abstract |
| Pollak, 2016 | Conference abstract |
| Pollak, 2017 | Conference abstract |
| Ponath, 2009 | Conference abstract |
| Raghavi, 2017 | Conference abstract |
| Reddy, 2013 | Conference abstract |
| Schwarz, 2006 | Conference abstract |
| Spalletta, 2015 | Conference abstract |
| Steiner, 2010 | Conference abstract |
| Taler, 2017 | Conference abstract |
| Tan, 2010b | Conference abstract |
| van de Kerkhof, 2009 | Conference abstract |
| van de Kerkhof, 2011a | Conference abstract |
| van de Kerkhof, 2011b | Conference abstract |
| van der Leeuw, 2013a | Conference abstract |
| Weickert, 2017 | Conference abstract |
| Weickert, 2018 | Conference abstract |
| Wong, 2010 | Conference abstract |
| Yanik, 2009 | Conference abstract |
| Zhang, 2010 | Conference abstract |
| Zincir, 2013 | Conference abstract |
| Kahlon, 2016 | Conference paper |
| Marenco, 2006 | Conference paper |
| Maxeiner, 2009 | Conference paper |
| Reiber, 2009 | Conference paper |
| Rothermundt, 2002 | Conference paper |
| Schwarz, 1999 | Conference paper |
| Ehrenreich, 2007 | Controls (blood donors) with unknown demographic and clinical information |
| Carrizoa, 2008 | Controls with family history of schizophrenia |
| Juchnowicz, 2021 | Data not available |
| Beumer, 2012 | Data not available |
| Deng, 2018 | Data not available |
| Domenici, 2010 | Data not available |
| Steinacker, 2021 | Data not available |
| Dencker, 1968 | Levels of biomarkers not quantified |
| Lasić, 2014 | Data not available |
| Gerner, 1982 | Data not available |
| Werner, 2022a | Data not available |
| Xiong, 2010 | Data not available |
| Xiong, 2014 | Likely inaccurate data with identical standard deviations across molecules; data not received |
| Kuehne, 2013 | Mixed affective disorders |
| Barcones, 2018 | Mixed affective psychosis |
| García-Bueno, 2014 | Mixed affective psychosis |
| García-Bueno, 2015 | Mixed affective psychosis |
| Johansson, 2017 | Mixed affective psychosis |
| Wedervang-Resell, 2020 | Mixed affective psychosis |
| Aas, 2020 | Mixed bipolar and schizoaffective disorder |
| Bioque, 2019 | Mixed psychiatric disorders |
| Chang, 2011 | No diagnostic tool or exclusion criteria applied |
| Graham, 2008 | No diagnostic tool specified |
| Parikh, 2003 | No diagnostic tool specified |
| Valiuliene, 2021 | No diagnostic tool specified |
| Aloe, 1997 | No healthy controls |
| Axelsson, 1982 | No healthy controls |
| Bălăiţă, 1984 | No healthy controls |
| Bauer, 1987 | No healthy controls |
| Bechter, 2010 | No healthy controls |
| Bourassa, 2020 | No healthy controls |
| Bullock, 1951 | No healthy controls |
| Chengappa, 2018 | No healthy controls |
| Chu, 2018a | No healthy controls |
| Chu, 2018b | No healthy controls |
| Chukaew, 2022 | No healthy controls |
| de-Oliveira, 2018 | No healthy controls |
| Ding, 2019 | No healthy controls |
| Doran, 1986 | No healthy controls |
| Doran, 1989 | No healthy controls |
| Dorofeikova, 2018 | No healthy controls |
| Endres, 2020 | No healthy controls |
| Endres, 2021 | No healthy controls |
| Falcone, 2015a | No healthy controls |
| Fawzi, 2015 | No healthy controls |
| Feng, 2020 | No healthy controls |
| Fitzgerald, 2003 | No healthy controls |
| Gannon, 2020 | No healthy controls |
| Heikkila, 1993 | No healthy controls |
| Hunter, 1969 | No healthy controls |
| Ishioka, 2015 | No healthy controls |
| Kirch, 1985 | No healthy controls |
| Klemettila, 2017 | No healthy controls |
| Krivoy, 2018 | No healthy controls |
| Linnoila, 1983 | No healthy controls |
| Margulska, 2018 | No healthy controls |
| Martínez-Pinteño, 2022 | No healthy controls |
| Meng, 1965 | No healthy controls |
| Morera-Fumero, 2021 | No healthy controls |
| Müller, 1995 | No healthy controls |
| Nikisch, 2012 | No healthy controls |
| Oviedo-Salcedo, 2021 | No healthy controls |
| Pedersen, 2008 | No healthy controls |
| Raposo, 2011 | No healthy controls |
| Räuber, 2021 | No healthy controls |
| Romash, 2021 | No healthy controls |
| Scheiber, 2022 | No healthy controls |
| Schwarz, 1998 | No healthy controls |
| Sharma, 1994 | No healthy controls |
| Sharma, 1995 | No healthy controls |
| Śmierciak, 2021 | No healthy controls |
| Stålberg, 2014 | No healthy controls |
| Strzelecki, 2016 | No healthy controls |
| Susai, 2022 | No healthy controls |
| Tomasik, 2016 | No healthy controls |
| Torrey, 1978 | No healthy controls |
| Tosato, 2020 | No healthy controls |
| Wahlbeck, 2000a | No healthy controls |
| Wahlbeck, 2000b | No healthy controls |
| Wu, 2018 | No healthy controls |
| Cameron, 1967 | No healthy controls; diagnostic tool unspecified |
| Banki, 1992a | Neurological controls (peripheral neuralgias, migraine, or musculoskeletal pain disorders) |
| Banki, 1992b | Neurological controls (migraine headache, musculoskeletal, or other neuralgias) |
| Delisi, 1981 | Neurological controls |
| Gjerris, 1984 | Neurological controls |
| Melkersson, 2018 | Neurological controls |
| Rafaelsen, 1985 | Psychiatric controls |
| Balõtšev, 2019 | Patients and a control with cannabis use |
| Haring, 2015 | Patients and a control with cannabis use |
| Lizano, 2016 | Patients with alcohol or cannabis use disorder |
| Lizano, 2021 | Patients and controls with cardiovascular disorders |
| Martinez-Cengotitabengoa, 2016 | Patients and controls with cannabis use |
| Mohite, 2018 | Patients with diabetes and hypertension |
| Schwarz, 2012 | Patients and controls with cannabis use |
| Chow, 2015 | Patients and controls with heart failure and stroke |
| Elkjaer Greenwood Ormerod, 2022 | Patients with substance use disorder |
| Jordan, 2018 | Patients with cannabis use |
| Rissler, 1986 | Patients with depression, cardiorespiratory insufficiency; controls treated with antidepressants |
| Werner, 2022b | Patients with infection and autoimmune diseases |
| Kirch, 1992 | Patients with substance abuse |
| Nishino, 1998 | Patients with substance abuse |
| van de Kerkhof, 2013 | Patients with substance abuse |
| van der Leeuw, 2013b | Patients with substance-induced psychotic disorder |
| van der Leeuw, 2017 | Patients with substance-induced psychotic disorder; controls with MDD |
| Jeppesen, 2022 | Patients and controls with neurological soft signs |
| Di Nicola, 2013 | Patients with unspecified physical health issues, sickle cell disease and asthma |
| Falcone, 2010 | Participants overlapped with Falcone, 2015 |
| Klaus, 2022 | Participants overlapped with Klaus, 2021 |
| Masopust, 2011 | Participants overlapped with Masopust, 2013 |
| Schwarz, 2000 | Participants overlapped with Krönig, 2005 |
| Steiner, 2012 | Participants overlapped with Steiner, 2006 and 2009 |
| XIao, 2019 | Participants overlapped with Xiao, 2018 |
| Xiong, 2011 | Participants same as Xiong, 2010 |
| Yamamori, 2013 | Participants overlapped with Kudo, 2020 |
| Ye, 2018 | Participants overlapped with Xiao, 2018 |
| Zhao, 2019 | Participants overlapped with Xiao, 2018 |
| Lindström, 1985 | Review |
| Tan, 2010a | Retracted |
| Jiang, 2003 | Measured other molecules |
| Solomon, 1969 | Measured serum Ig |
| Herberth, 2014 | Used blood cell culture |

Table S6. Summary of the molecules examined in 1 study

| **Molecule** | **Tissue** | **Study** | **Patients** | | |  | **Controls** | | | ***P*** |
| --- | --- | --- | --- | --- | --- | --- | --- | --- | --- | --- |
|  |  |  | **N** | **Mean** | **SD** |  | **N** | **Mean** | **SD** |  |
| Cadherin 2 (N-cadherin) | Plasma | Sheikh, 2023 | 895 | 6.96 | 1.79 |  | 1070 | 6.72 | 1.64 | NS |
| Cadherin 5 (VE-cadherin) | Serum | Smirnova, 2019 | 33 | 2.84 | 3.87 |  | 24 | 3.19 | 2.31 | 0.55 |
| Claudin | Serum | Yazla, 2022 | 75 | 9.45 | 11.19 |  | 41 | 8.26 | 7.50 | 0.804 |
| Claudin 5 | Serum | Usta, 2021 | 50 | 959.80 | 583.00 |  | 50 | 1230.70 | 692.40 | **0.037** |
| Fibrinogen | CSF | Hayes, 2014 | 46 | 0.19 | 0.22 |  | 35 | 0.22 | 0.17 | **0.021** |
| IgG index | CSF | Severance, 2015 | 54 | 0.48 | 0.06 |  | 60 | 0.47 | 0.08 | NS |
| IgG ratio | CSF:serum | Severance, 2015 | 54 | 2.63 | 1.20 |  | 60 | 1.95 | 0.77 | **drug-naïve ≤ 0.002; medicated ≤ 0.001** |
| JAM-A | Plasma | Sheikh, 2023 | 895 | 1.31 | 0.55 |  | 1070 | 1.20 | 0.50 | **<0.001** |
| MMP-1 | Serum | Tylec, 2021 | 25 | 1.52 | 0.57 |  | 25 | 1.25 | 0.68 | NS |
| MMP-2 | Serum | Shibasaki, 2016 | 13 | 171.55 | 55.53 |  | 40 | 188.06 | 27.78 | NS |
| MMP-2 | CSF | Omori, 2020 | 86 | 55.90 | 18.90 |  | 106 | 49.80 | 18.10 | **0.031** |
| MMP-3 | CSF | Hayes, 2014 | 46 | 0.17 | 0.07 |  | 35 | 0.16 | 0.07 | 0.647 |
| MMP-7 | Serum | Tylec, 2021 | 25 | 1.22 | 1.05 |  | 25 | 2.29 | 0.28 | **<0.001** |
| MMP-13 | Serum | Tylec, 2021 | 25 | 0.84 | 0.30 |  | 25 | 0.83 | 0.13 | NS |
| NGF | CSF | Kale, 2009 | 28 | 17.13 | 13.34 |  | 14 | 39.20 | 39.40 | **0.038** |
| Occludin | Serum | Yazla, 2022 | 75 | 14.97 | 8.50 |  | 41 | 20.81 | 14.63 | 0.058 |
| PGE | CSF | Mathe, 1980 | 8 | 2894.00 | 1374.62 |  | 9 | 862.00 | 267.00 | **<0.001** |

Table S7. Bias assessment according to the adapted version of NOS

| **Study** | **Selection** | | | |  | **Comparability** | |  | **Exposure** | | **Total** |
| --- | --- | --- | --- | --- | --- | --- | --- | --- | --- | --- | --- |
|  | **1** | **2** | **3** | **4** |  | **1** | **2** |  | **1** | **2** |  |
| Ajami, 2014 | 0 | 1 | 1 | 1 |  | 1 | 1 (sex) |  | 1 | 1 | 7 |
| Ali, 2017 | 1 | 1 | 1 | 1 |  | 1 | 1 (sex, smoking) |  | 0 | 1 | 7 |
| Arabska, 2019 | 0 | 0 | 0 | 1 |  | 1 | 1 (BMI, FMI, blood pressure, etc.) |  | 0 | 1 | 4 |
| Balõtšev, 2017 | 1 | 1 | 1 | 0 |  | 0 | 0 |  | 0 | 1 | 4 |
| Bersani, 2004 | 0 | 1 | 0 | 0 |  | 0 | 0 |  | 1 | 1 | 3 |
| Bissette, 1986 | 0 | 1 | 0 | 0 |  | 0 | 0 |  | 0 | 1 | 2 |
| Bocchio-Chiavetto, 2018 | 1 | 1 | 1 | 1 |  | 0 | 1 (sex, BMI, age at onset) |  | 0 | 1 | 6 |
| Cai, 2020 | 1 | 1 | 0 | 1 |  | 0 | 0 |  | 0 | 1 | 4 |
| Chen, 2017a | 1 | 1 | 1 | 1 |  | 1 | 0 |  | 1 | 1 | 7 |
| Chen, 2017b | 1 | 1 | 1 | 1 |  | 0 | 0 |  | 0 | 1 | 5 |
| Chenniappan, 2022 | 1 | 1 | 0 | 0 |  | 1 | 1 (sex) |  | 0 | 1 | 5 |
| Dai, 2020 | 0 | 1 | 1 | 1 |  | 0 | 0 |  | 0 | 1 | 4 |
| Devanarayanan, 2016 | 0 | 1 | 1 | 0 |  | 0 | 0 |  | 0 | 1 | 3 |
| Elmi, 2019 | 0 | 1 | 1 | 1 |  | 1 | 1 (sex, race) |  | 0 | 1 | 6 |
| Falcone, 2015 | 1 | 0 | 0 | 1 |  | 0 | 0 |  | 0 | 1 | 3 |
| Gattaz, 2000 | 0 | 1 | 0 | 0 |  | 0 | 0 |  | 0 | 1 | 2 |
| Gerasimou, 2018 | 0 | 1 | 1 | 0 |  | 0 | 0 |  | 0 | 1 | 3 |
| Gerner, 1985 | 0 | 1 | 0 | 0 |  | 0 | 0 |  | 0 | 1 | 2 |
| Goff, 2018 | 1 | 1 | 1 | 1 |  | 1 | 1 (sex, education) |  | 0 | 1 | 7 |
| Gokulakrishnan, 2022 | 1 | 1 | 1 | 0 |  | 0 | 0 |  | 0 | 1 | 4 |
| Hayes, 2014 | 0 | 1 | 1 | 1 |  | 0 | 0 |  | 0 | 1 | 4 |
| Hendouei, 2016 | 1 | 1 | 0 | 1 |  | 1 | 1 (sex) |  | 0 | 1 | 6 |
| Hidese, 2020 | 1 | 1 | 1 | 0 |  | 1 | 1 (sex, ethnicity) |  | 0 | 1 | 6 |
| Hong, 2016 | 1 | 1 | 1 | 0 |  | 1 | 1 (sex) |  | 0 | 1 | 6 |
| Huang, 2022 | 0 | 1 | 1 | 1 |  | 1 | 1 (sex, BMI, # of cigarettes/day) |  | 0 | 1 | 6 |
| Iwata, 2007 | 0 | 1 | 1 | 1 |  | 1 | 1 (sex, WBC count) |  | 0 | 1 | 6 |
| Jockers-Scherubl, 2003 | 0 | 0 | 0 | 0 |  | 0 | 0 |  | 0 | 1 | 1 |
| Jockers-Scherubl, 2006 | 0 | 0 | 0 | 0 |  | 0 | 0 |  | 0 | 1 | 1 |
| Kale, 2009 | 1 | 1 | 1 | 1 |  | 1 | 1 (sex, socioeconomic status) |  | 1 | 1 | 8 |
| Kannan, 2017 | 1 | 1 | 1 | 1 |  | 0 | 0 |  | 0 | 1 | 5 |
| Kavzoglu, 2013 | 0 | 1 | 0 | 1 |  | 1 | 1 (sex, smoking, BMI) |  | 0 | 1 | 5 |
| Keshri, 2021 | 0 | 1 | 1 | 1 |  | 1 | 1 (sex) |  | 0 | 1 | 6 |
| Klaus, 2021 | 0 | 1 | 1 | 1 |  | 0 | 0 |  | 0 | 1 | 4 |
| Kozłowska, 2021 | 0 | 0 | 1 | 1 |  | 0 | 1 (sex) |  | 0 | 1 | 4 |
| Krönig, 2005 | 1 | 1 | 0 | 1 |  | 0 | 0 |  | 1 | 1 | 5 |
| Kudo, 2019 | 1 | 1 | 1 | 1 |  | 1 | 1 (sex) |  | 0 | 1 | 7 |
| Lara, 2001 | 0 | 1 | 1 | 1 |  | 1 | 1 (sex) |  | 0 | 1 | 6 |
| Lee, 2009 | 0 | 1 | 1 | 1 |  | 1 | 1 (sex) |  | 0 | 1 | 6 |
| Lee, 2015 | 0 | 1 | 1 | 1 |  | 0 | 1 (BMI, smoking) |  | 0 | 1 | 5 |
| Lin, 2018 | 1 | 1 | 1 | 1 |  | 1 | 1 (sex, BMI) |  | 0 | 1 | 7 |
| Ling, 2007 | 1 | 1 | 1 | 1 |  | 0 | 0 |  | 0 | 1 | 5 |
| Liu, 2020 | 1 | 1 | 1 | 1 |  | 1 | 1 (sex) |  | 1 | 1 | 8 |
| Liu, 2021 | 1 | 1 | 1 | 1 |  | 0 | 0 |  | 1 | 1 | 6 |
| Masopust, 2013 | 1 | 1 | 1 | 1 |  | 1 | 1 (sex, weight, BMI) |  | 0 | 1 | 7 |
| Mathe, 1980 | 0 | 0 | 0 | 0 |  | 1 | 1 (sex) |  | 1 | 1 | 4 |
| Milleit, 2016 | 1 | 1 | 0 | 1 |  | 1 | 1 (sex) |  | 0 | 1 | 6 |
| Mohite, 2017 | 0 | 1 | 1 | 0 |  | 1 | 1 (sex, race, education) |  | 0 | 1 | 5 |
| Morera-Fumero, 2017 | 1 | 1 | 0 | 1 |  | 1 | 1 (sex) |  | 1 | 1 | 7 |
| Morera-Fumero, 2022 | 1 | 1 | 1 | 1 |  | 1 | 1 (sex, season of admission) |  | 1 | 1 | 8 |
| Murphy, 2014 | 1 | 1 | 0 | 1 |  | 1 | 1 (sex) |  | 0 | 1 | 6 |
| Neugebauer, 2019 | 0 | 1 | 0 | 1 |  | 1 | 1 (sex) |  | 0 | 1 | 5 |
| Nguyen, 2018 | 0 | 1 | 1 | 1 |  | 0 | 1 (sex, race) |  | 1 | 1 | 6 |
| Niitsu, 2014 | 1 | 1 | 1 | 1 |  | 1 | 1 (sex) |  | 0 | 1 | 7 |
| O'Connell, 2013 | 0 | 0 | 1 | 1 |  | 0 | 1 (sex, BMI) |  | 0 | 1 | 4 |
| Omori, 2020 | 1 | 1 | 1 | 1 |  | 1 | 1 (sex) |  | 0 | 1 | 7 |
| Peters, 1990 | 0 | 0 | 0 | 0 |  | 1 | 1 (sex) |  | 0 | 1 | 3 |
| Pillai, 2017 | 0 | 1 | 1 | 1 |  | 0 | 0 |  | 1 | 1 | 5 |
| Qi, 2009 | 0 | 1 | 1 | 1 |  | 1 | 1 (sex) |  | 1 | 1 | 7 |
| Qing, 2021 | 0 | 1 | 1 | 1 |  | 0 | 0 |  | 0 | 1 | 4 |
| Radu, 2020 | 0 | 1 | 0 | 1 |  | 1 | 1 (sex, ethnicity, smoking, BMI) |  | 0 | 1 | 5 |
| Reinikainen, 1990 | 0 | 1 | 0 | 1 |  | 0 | 0 |  | 0 | 1 | 3 |
| Roos, 1985 | 0 | 1 | 0 | 0 |  | 0 | 0 |  | 0 | 1 | 2 |
| Rothermundt, 2001 | 1 | 0 | 0 | 1 |  | 1 | 1 (sex) |  | 0 | 1 | 5 |
| Rothermundt, 2004 | 1 | 1 | 0 | 1 |  | 1 | 1 (sex) |  | 0 | 1 | 6 |
| Rothermundt, 2007 | 0 | 0 | 0 | 1 |  | 1 | 1 (sex) |  | 0 | 1 | 4 |
| Rubinow, 1986 | 0 | 1 | 0 | 1 |  | 1 | 1 (sex) |  | 0 | 1 | 5 |
| Sarandol, 2007 | 0 | 0 | 0 | 1 |  | 1 | 1 (sex, smoking) |  | 0 | 1 | 4 |
| Schmitt, 2005 | 0 | 0 | 0 | 1 |  | 1 | 1 (sex) |  | 0 | 1 | 4 |
| Schroeter, 2003 | 1 | 0 | 0 | 1 |  | 1 | 0 |  | 0 | 1 | 4 |
| Schroeter, 2009 | 1 | 0 | 0 | 1 |  | 1 | 1 (sex) |  | 0 | 1 | 5 |
| Schwarz, 1998 | 1 | 0 | 0 | 0 |  | 1 | 1 (sex) |  | 1 | 1 | 5 |
| Seitz-Holland, 2022 | 0 | 1 | 1 | 1 |  | 1 | 1 (sex) |  | 0 | 1 | 6 |
| Severance, 2015 | 0 | 0 | 0 | 0 |  | 0 | 0 |  | 0 | 1 | 1 |
| Sheikh, 2023 | 0 | 1 | 1 | 1 |  | 1 | 1 (sex, BMI, CRP) |  | 0 | 0 | 5 |
| Shibasaki, 2016 | 0 | 1 | 0 | 1 |  | 0 | 0 |  | 0 | 1 | 3 |
| Smirnova, 2019 | 0 | 1 | 0 | 1 |  | 1 | 1 (sex) |  | 0 | 1 | 5 |
| Stefanovic, 2016 | 1 | 1 | 1 | 1 |  | 1 | 1 (sex, BMI, smoking) |  | 1 | 1 | 8 |
| Steiner, 2006 | 0 | 0 | 0 | 1 |  | 1 | 1 (sex) |  | 0 | 1 | 4 |
| Steiner, 2009 | 0 | 0 | 0 | 0 |  | 1 | 1 (sex) |  | 0 | 1 | 3 |
| Turkmen, 2021 | 1 | 1 | 0 | 1 |  | 0 | 0 |  | 0 | 1 | 4 |
| Tylec, 2021 | 1 | 0 | 0 | 1 |  | 1 | 0 |  | 0 | 1 | 4 |
| Usta, 2021 | 0 | 1 | 1 | 1 |  | 0 | 0 |  | 0 | 1 | 4 |
| Uzbay, 2013 | 0 | 1 | 0 | 1 |  | 0 | 0 |  | 0 | 1 | 3 |
| Wahlbeck, 1993 | 0 | 0 | 1 | 1 |  | 1 | 0 |  | 0 | 1 | 4 |
| Wahlbeck, 1997 | 0 | 1 | 1 | 1 |  | 1 | 1 (sex) |  | 0 | 1 | 6 |
| Wahlbeck, 1998 | 0 | 1 | 1 | 1 |  | 0 | 0 |  | 0 | 1 | 4 |
| Widerlov, 1988 | 1 | 0 | 0 | 1 |  | 0 | 0 |  | 0 | 1 | 3 |
| Wiesmann, 1999 | 1 | 0 | 0 | 0 |  | 1 | 1 (sex) |  | 0 | 1 | 4 |
| Xiao, 2018 | 1 | 1 | 0 | 0 |  | 1 | 1 (sex) |  | 1 | 1 | 6 |
| Yazla, 2022 | 0 | 1 | 1 | 1 |  | 0 | 0 |  | 0 | 1 | 4 |
| Zakharyan, 2014 | 1 | 1 | 1 | 1 |  | 1 | 1 (sex) |  | 0 | 1 | 7 |
| Zhang, 2010a | 1 | 0 | 1 | 0 |  | 1 | 1 (sex) |  | 1 | 1 | 6 |
| Zhang, 2010b | 1 | 1 | 1 | 1 |  | 1 | 1 (sex, smoking) |  | 1 | 1 | 8 |

BMI, body mass index. BP, blood pressure. CRP, C-reactive protein. FMI, fat mass index. WBC, white blood cell.

Figure S1. Contour-enhanced funnel plots of molecules examined in ≥10 studies

Table S8. Egger test results of molecules examined in ≥10 studies

|  | **β1** | **SE of β1** | ***Z*** | ***P*** |
| --- | --- | --- | --- | --- |
| NGF | 0.340 | 6.403 | 0.050 | 0.958 |
| S100B | 2.370 | 1.713 | 1.380 | 0.167 |
| VEGF | 0.880 | 1.962 | 0.450 | 0.653 |

Figure S2. Galbraith plots of the molecules with outlier studies

Figure S3. Bubble plots of blood S100B

Table S9. Treatment regimens regarding molecules examined in ≥5 studies

| **Study** | **Number of patients** | **Medications** | **Treatment duration** | **Dosing (mg/day)** | | |
| --- | --- | --- | --- | --- | --- | --- |
|  |  |  |  | **Original** | **CPZeq** | **DDD** |
| **Blood ICAM-1** |  |  |  |  |  |  |
| Cai, 2020 | 78 | AP, AD, MS, BDZ (multiple types; pct NA; some on combi-therapy) | ≥1 year | NA | 568.6 ± 488.8 |  |
| Kavzoglu, 2013 | 39 | NA | NA | NA |  |  |
| Nguyen, 2018 | 99 | AP (types & pct NA) | NA | NA |  | 1.7 ± 1.5 |
| Radu, 2020 | 50 | 60.0% olanzapine, 40.0% risperidone | 6 months | Olanzapine: 10; risperidone: 6 |  |  |
| Schwarz, 1998 | 24 | Haloperidol, clozapine, olanzapine (pct NA) | 0.5–7 months | NA |  |  |
| **Blood MMP-9** |  |  |  |  |  |  |
| Chenniappan, 2022 | 62 | NA | NA | NA |  |  |
| Niitsu, 2014 | 63 | 14.3% aripiprazole, 28.6% olanzapine, 12.7% quetiapine, 39.7% risperidone | ≥8 weeks | NA | 323.9 ± 184.2 (n=60) |  |
| Seitz-Holland, 2022 | 30 | AP (types & pct NA) | NA | NA | 740.8 ± 385.1 |  |
| Shibasaki, 2016 | 13 | 53.8% risperidone, 30.8% olanzapine, 30.8% quetiapine, 23.1% zotepine, 15.4% blonanserin, 7.7% aripiprazole, 7.7% haloperidol, 7.7% chlorpromazine, 23.1% i.v. haloperidol | NA | Risperidone: 1.5–12; olanzapine: 10–20; quetiapine: 300–675; zotepine: 150–400; blonanserin: 12–24; aripiprazole: 24; haloperidol: 30; chlorpromazine: 450; i.v. haloperidol: 5–10 |  |  |
| **Blood NGF** |  |  |  |  |  |  |
| Ajami, 2014 | 26 | 38.5% clozapine, 61.5% risperidone | 40 days | NA |  |  |
| Jockers-Scherubl, 2006 | 66 | NA | ≥4 weeks | NA |  |  |
| Lee, 2009 | 36 | 100.0% risperidone | 6 weeks | 5.7 ± 2.0 |  |  |
| Neugebauer, 2019 | 18 | AP (types & pct NA; 44.0% combi-therapy) | NA | NA | 664.2 ± 358.2 |  |
| Zakharyan, 2014 | 120 | 100.0% haloperidol | NA | NA |  |  |
| **Blood S100B** |  |  |  |  |  |  |
| Gattaz, 2000 | 23 | 69.6% clozapine | NA | NA | 710.0 ± 340.0 |  |
| Gerasimou, 2018 | 13 | 100.0% olanzapine | 6 weeks | NA |  |  |
| Goff, 2018 | 27 | AP (types & pct NA) | NA | NA |  |  |
| Hendouei, 2016 | 19 | 31.6% clozapine, 68.4% risperidone | ≥6 months | NA | 345.0 ± 5.0 |  |
| Kannan, 2017 | 175 | NA | NA | NA |  |  |
| Kozłowska, 2021 | 58 | NA | NA | NA | 746.5 ± 642.2 | 2.5 ± 1.1 |
| Ling, 2007 | 57 | 17.5% clozapine, other types & pct NA | 12 weeks | NA |  |  |
| Morera-Fumero, 2017 | 65 | AP (types & pct NA; most on combi-therapy) | NA | NA | 757.7 ± 400.0 |  |
| Morera-Fumero, 2022 | 23 | NA | NA | NA | 811.6 ± 406.7 |  |
| O'Connell, 2013 | 97 | 100.0% clozapine | 7.0 ± 4.6 years | 432.1 ± 185.7 | 360.1 ± 154.8 |  |
| Qi, 2009 | 63 | 58.7% clozapine, 19.1% haloperidol, 7.9% chlorpromazine, 7.9% perphenazine, 6.4% other typical AP | 46.8 ± 60.6 months | NA | 458.0 ± 529.0 |  |
| Rothermundt, 2001 | 26 | 15.4% bromperidole, 11.5% ﬂuphenazine, 11.5% benperidole, 11.5% haloperidole, 3.9% ﬂupenthixole, 15.4% clozapin, 11.5% olanzapine, 19.2% risperidone | 6 weeks | NA |  |  |
| Rothermundt, 2004 | 98 | 54.1% risperidone, 45.9% flupenthixol, BDZ/AC/NBDZ-hypnotics (types & pct NA) | 12 weeks | Flupenthixol: 4–12; risperidone: 2–6 |  |  |
| Sarandol, 2007 | 36 | Risperidone, olanzapine, clozapine, quetiapine, amisulpride, haloperidol, long-acting risperidone (pct NA) | 6 weeks | NA |  |  |
| Schmitt, 2005 | 41 | 34.1% typical AP, 36.6% clozapine, 19.5% haloperidol + typical AP + clozapine | 27.2 ± 12.3 years | NA | 708.5 ± 591.7 |  |
| Schroeter, 2003 | 16 | Flupentixol, fluphenazine, haloperidol, perazine, pimozide, zuclopenthixol, amisulpiride, clozapine, olanzapine, risperidone, sulpiride (pct NA) | NA | NA | 363.1 ± 248.8 |  |
| Schroeter, 2009 | 16 | NA | NA | NA |  |  |
| Steiner, 2006 | 12 | NA | NA | NA |  |  |
| Steiner, 2009 | 26 | Amisulpride, aripiprazole, clozapine, olanzapine, quetiapine, risperidone, ziprasidone (pct NA) | ≥6 weeks | NA |  |  |
| Wiesmann, 1999 | 20 | NA | NA | NA |  |  |
| Zhang, 2010 | 50 | 40.0% clozapine, 28.0% risperidone, 10.0% perphenazine, 6.0% chlorpromazine, 8.0% haloperidol, 8.0% sulpiride | ≥12 months | NA | 419.0 ± 471.0 |  |
| Zhang, 2010 | 82 | 37.8% clozapine, 26.8% risperidone, 11.0% perphenazine, 9.8% haloperidol, 7.3% chlorpromazine, 7.3% sulpiride | ≥12 months | NA |  |  |
| **Blood VCAM-1** |  |  |  |  |  |  |
| Kavzoglu, 2013 | 39 | NA | NA | NA |  |  |
| Nguyen, 2018 | 99 | AP (types & pct NA) | NA | NA |  | 1.7 ± 1.5 |
| Radu, 2020 | 50 | 60.0% olanzapine, 40.0% risperidone | 6 months | Olanzapine: 10; risperidone: 6 |  |  |
| **Blood VEGF** |  |  |  |  |  |  |
| Balõtšev, 2017 | 105 | 28.6% haloperidol, 27.6% zuclopenthixol, 22.9% clozapine, 16.2% chlorprothixene, 12.4% risperidone, 10.5% olanzapine, 5.7% aripiprazole, 5.7% sertindole, 4.8% quetiapine, 4.8% sulpiride, 2.9% perphenazine, 1.9% melperone, 1.9% chlorpromazine, 1.0% ﬂupenthixol | ≥1 month | NA |  |  |
| Lee, 2015 | 50 | 28.0% amisulpride, 48.0% risperidone, 14.0% aripiprazole, 6.0% quetiapine, 4.0% olanzapine | 6 weeks | NA | 433.5 ± 211.4 |  |
| Liu, 2020 | 165 | NA | NA | NA | 593.9 ± 803.4 |  |
| Liu, 2021 | 138 | NA | ≥3 months | NA |  |  |
| Murphy, 2014 | 15 | 100.0% quetiapine | 12 weeks | 200 (n=5)/400 (n=10) for 4 weeks → doses adjusted for 8 weeks |  |  |
| Nguyen, 2018 | 99 | AP (types & pct NA) | NA | NA |  | 1.7 ± 1.5 |
| Pillai, 2017 | 96 | AP (69.8% monotherapy, 30.2% combination therapy) | ≥1 year | NA | 541.6 ± 457.8 |  |
| Xiao, 2018 | 118 | 23.7% risperidone, 20.3% quetiapine, 21.2% olanzapine, 19.5% aripiprazole, 15.3% ziprasidone | 6 weeks | Risperidone: 3–6; quetiapine: 300–600; olanzapine: 10–20; aripiprazole: 10–20; ziprasidone: 20–60 |  |  |

Table shows mean ± standard deviation. AC, anticholinergics. AD, antidepressants. AP, antipsychotics. APKS, antiparkinsonisms. BDZ, benzodiazepines. CPZeq, chlorpromazine equivalents. DDD, defined daily dose. ICAM-1, intercellular adhesion molecule 1. MMP-9, matrix metalloproteinase-9. MS, mood stabilizers. NA, not available. NBDZ, non-benzodiazepine. NGF, nerve growth factor. Pct, percentage. VCAM-1, vascular cell adhesion molecule 1. VEGF, vascular endothelial growth factor.

References

1. Ajami A, Hosseini SH, Taghipour M, Khalilian A (2014): Changes in serum levels of brain derived neurotrophic factor and nerve growth factor-beta in schizophrenic patients before and after treatment. *Scand J Immunol*. 80:36-42.

2. Ali FT, Abd El-Azeem EM, Hamed MA, Ali MAM, Abd Al-Kader NM, Hassan EA (2017): Redox dysregulation, immuno-inflammatory alterations and genetic variants of BDNF and MMP-9 in schizophrenia: Pathophysiological and phenotypic implications. *Schizophrenia Research*. 188:98-109.

3. Arabska J, Margulska A, Strzelecki D, Wysokinski A (2019): Does metabolic status affect serum levels of BDNF and MMP-9 in patients with schizophrenia? *Nordic Journal of Psychiatry*. 73:515-521.

4. Balotsev R, Koido K, Vasar V, Janno S, Kriisa K, Mahlapuu R, et al. (2017): Inflammatory, cardio-metabolic and diabetic profiling of chronic schizophrenia. *European Psychiatry*. 39:1-10.

5. Bersani G, Iannitelli A, Massoni E, Garavini A, Grilli A, Di Giannantonio M, et al. (2004): Ultradian variation of nerve growth factor plasma levels in healthy and schizophrenic subjects. *Int J Immunopathol Pharmacol*. 17:367-372.

6. Bissette G, Widerlöv E, Walléus H, Karlsson I, Eklund K, Forsman A, et al. (1986): Alterations in cerebrospinal fluid concentrations of somatostatinlike immunoreactivity in neuropsychiatric disorders. *Arch Gen Psychiatry*. 43:1148-1151.

7. Bocchio-Chiavetto L, Zanardini R, Tosato S, Ventriglia M, Ferrari C, Bonetto C, et al. (2018): Immune and metabolic alterations in first episode psychosis (FEP) patients. *Brain Behavior and Immunity*. 70:315-324.

8. Cai HQ, Catts VS, Webster MJ, Galletly C, Liu D, O'Donnell M, et al. (2020): Increased macrophages and changed brain endothelial cell gene expression in the frontal cortex of people with schizophrenia displaying inflammation. *Molecular Psychiatry*. 25:761-775.

9. Chen S, Tian L, Chen N, Xiu MH, Wang ZR, Yang GG, et al. (2017): Cognitive dysfunction correlates with elevated serum S100B concentration in drug-free acutely relapsed patients with schizophrenia. *Psychiatry Research*. 247:6-11.

10. Chen SZ, Jiang HT, Liu Y, Hou ZH, Yue YY, Zhang YQ, et al. (2017): Combined serum levels of multiple proteins in tPA-BDNF pathway may aid the diagnosis of five mental disorders. *Scientific Reports*. 7:9.

11. Chenniappan R, Nandeesha H, Kattimani S, Goud AC, Thiagarajan D (2022): Risperidone Reduces Matrix Metalloproteinase-9 and Increases Neurotrophin-3 in Schizophrenia Spectrum of Disorder. *Indian Journal of Clinical Biochemistry*.7.

12. Dai N, Jie HJ, Duan Y, Xiong P, Xu XF, Chen P, et al. (2020): Different serum protein factor levels in first-episode drug-naive patients with schizophrenia characterized by positive and negative symptoms. *Psychiatry and Clinical Neurosciences*. 74:472-479.

13. Devanarayanan S, Nandeesha H, Kattimani S, Sarkar S (2016): Relationship between matrix metalloproteinase-9 and oxidative stress in drug-free male schizophrenia: a case control study. *Clinical Chemistry and Laboratory Medicine*. 54:447-452.

14. Elmi S, Sahu G, Malavade K, Jacob T (2019): Role of tissue plasminogen activator and plasminogen activator inhibitor as potential biomarkers in psychosis. *Asian Journal of Psychiatry*. 43:105-110.

15. Falcone T, Janigro D, Lovell R, Simon B, Brown CA, Herrera M, et al. (2015): S100B blood levels and childhood trauma in adolescent inpatients. *Journal of Psychiatric Research*. 62:14-22.

16. Gattaz WF, Lara DR, Elkis H, Portela LV, Gonçalves CA, Tort AB, et al. (2000): Decreased S100-beta protein in schizophrenia: preliminary evidence. *Schizophr Res*. 43:91-95.

17. Gerasimou C, Tsoporis JN, Siafakas N, Hatziagelaki E, Kallergi M, Chatziioannou SN, et al. (2018): A Longitudinal Study of Alterations of S100B, sRAGE and Fas Ligand in Association to Olanzapine Medication in a Sample of First Episode Patients with Schizophrenia. *Cns & Neurological Disorders-Drug Targets*. 17:383-388.

18. Gerner RH, Vankammen DP, Ninan PT (1985): Cerebrospinal-Fluid Cholecystokinin, Bombesin and Somatostatin in Schizophrenia and Normals. *Progress in Neuro-Psychopharmacology & Biological Psychiatry*. 9:73-82.

19. Goff DC, Zeng B, Ardekani BA, Diminich ED, Tang Y, Fan X, et al. (2018): Association of Hippocampal Atrophy With Duration of Untreated Psychosis and Molecular Biomarkers During Initial Antipsychotic Treatment of First-Episode Psychosis. *JAMA Psychiatry*. 75:370-378.

20. Gokulakrishnan K, Nikhil J, Vs S, Holla B, Thirumoorthy C, Sandhya N, et al. (2022): Altered Intestinal Permeability Biomarkers in Schizophrenia: A Possible Link with Subclinical Inflammation. *Ann Neurosci*. 29:151-158.

21. Hayes LN, Severance EG, Leek JT, Gressitt KL, Rohleder C, Coughlin JM, et al. (2014): Inflammatory Molecular Signature Associated With Infectious Agents in Psychosis. *Schizophrenia Bulletin*. 40:963-972.

22. Hendouei N, Hosseini SH, Panahi A, Khazaeipour Z, Barari F, Sahebnasagh A, et al. (2016): Negative Correlation between Serum S100B and Leptin Levels in Schizophrenic Patients During Treatment with Clozapine and Risperidone: Preliminary Evidence. *Iranian Journal of Pharmaceutical Research*. 15:323-330.

23. Hidese S, Hattori K, Sasayama D, Tsumagari T, Miyakawa T, Matsumura R, et al. (2020): Cerebrospinal fluid neuroplasticity-associated protein levels in patients with psychiatric disorders: a multiplex immunoassay study. *Translational Psychiatry*. 10:13.

24. Hong W, Zhao M, Li HZ, Peng FL, Wang F, Li NN, et al. (2016): Higher Plasma S100B Concentrations in Schizophrenia Patients, and Dependently Associated with Inflammatory Markers. *Scientific Reports*. 6:8.

25. Huang Z, Kang M, Li G, Xiong P, Chen H, Kang L, et al. (2022): Predictive effect of Bayes discrimination in the level of serum protein factors and cognitive dysfunction in schizophrenia. *J Psychiatr Res*. 151:539-545.

26. Iwata Y, Suzuki K, Nakamura K, Matsuzaki H, Sekine Y, Tsuchiya KJ, et al. (2007): Increased levels of serum soluble L-selectin in unmedicated patients with schizophrenia. *Schizophrenia Research*. 89:154-160.

27. Jockers-Scherübl MC, Matthies U, Danker-Hopfe H, Lang UE, Mahlberg R, Hellweg R (2003): Chronic cannabis abuse raises nerve growth factor serum concentrations in drug-naive schizophrenic patients. *J Psychopharmacol*. 17:439-445.

28. Jockers-Scherübl MC, Rentzsch J, Danker-Hopfe H, Radzei N, Schürer F, Bahri S, et al. (2006): Adequate antipsychotic treatment normalizes serum nerve growth factor concentrations in schizophrenia with and without cannabis or additional substance abuse. *Neurosci Lett*. 400:262-266.

29. Kale A, Joshi S, Pillai A, Naphade N, Raju M, Nasrallah H, et al. (2009): Reduced cerebrospinal fluid and plasma nerve growth factor in drug-naïve psychotic patients. *Schizophr Res*. 115:209-214.

30. Kannan G, Gressitt KL, Yang S, Stallings CR, Katsafanas E, Schweinfurth LA, et al. (2017): Pathogen-mediated NMDA receptor autoimmunity and cellular barrier dysfunction in schizophrenia. *Translational Psychiatry*. 7:8.

31. Kavzoglu SO, Hariri AG (2013): Intracellular Adhesion Molecule (ICAM-1), Vascular Cell Adhesion Molecule (VCAM-1) and E-Selectin Levels in First Episode Schizophrenic Patients. *Klinik Psikofarmakoloji Bulteni-Bulletin of Clinical Psychopharmacology*. 23:205-214.

32. Keshri N, Nandeesha H, Rajappa M, Menon V (2021): Matrix metalloproteinase-9 increases the risk of cognitive impairment in schizophrenia. *Nordic Journal of Psychiatry*. 75:130-134.

33. Klaus F, Mitchell K, Liou SC, Eyler LT, Nguyen TT (2021): Chemokine MCP1 is associated with cognitive flexibility in schizophrenia: A preliminary analysis. *Journal of Psychiatric Research*. 138:139-145.

34. Kozlowska E, Brzezinska-Blaszczyk E, Agier J, Wysokinski A, Zelechowska P (2021): Alarmins (IL-33, sST2, HMGB1, and S100B) as potential biomarkers for schizophrenia. *Journal of Psychiatric Research*. 138:380-387.

35. Kronig H, Riedel M, Schwarz MJ, Strassnig M, Moller HJ, Ackenheil M, et al. (2005): ICAM G241A polymorphism and soluble ICAM-1 serum levels: Evidence for an active immune process in schizophrenia. *Neuroimmunomodulation*. 12:54-59.

36. Kudo N, Yamamori H, Ishima T, Nemoto K, Yasuda Y, Fujimoto M, et al. (2020): Plasma levels of matrix metalloproteinase-9 (MMP-9) are associated with cognitive performance in patients with schizophrenia. *Neuropsychopharmacol Rep*. 40:150-156.

37. Lara DR, Gama CS, Belmonte-de-Abreu P, Portela LVC, Goncalves CA, Fonseca M, et al. (2001): Increased serum S100B protein in schizophrenia: a study in medication-free patients. *Journal of Psychiatric Research*. 35:11-14.

38. Lee BH, Kim YK (2009): Increased plasma brain-derived neurotropic factor, not nerve growth factor-Beta, in schizophrenia patients with better response to risperidone treatment. *Neuropsychobiology*. 59:51-58.

39. Lee BH, Hong JP, Hwang JA, Ham BJ, Na KS, Kim WJ, et al. (2015): Alterations in plasma vascular endothelial growth factor levels in patients with schizophrenia before and after treatment. *Psychiatry Research*. 228:95-99.

40. Lin YZ, Peng YM, He S, Xu JJ, Shi Y, Su YS, et al. (2018): Serum IL-1ra, a novel biomarker predicting olanzapine-induced hypercholesterolemia and hyperleptinemia in schizophrenia. *Progress in Neuro-Psychopharmacology & Biological Psychiatry*. 84:71-78.

41. Ling SH, Tang YL, Jiang F, Wiste A, Guo SS, Weng YZ, et al. (2007): Plasma S-100B protein in Chinese patients with schizophrenia: Comparison with healthy controls and effect of antipsychotics treatment. *Journal of Psychiatric Research*. 41:36-42.

42. Liu YH, Huang LZ, Chen JX, Tan SP, Zhao KX, Yan SX, et al. (2020): Retinal venule correlation with schizophrenia. *International Journal of Clinical and Experimental Medicine*. 13:6927-6935.

43. Liu YH, Chen JX, Huang L, Yan SX, Bian QT, Yang FD (2021): Relationships Among Retinal Nerve Fiber Layer Thickness, Vascular Endothelial Growth Factor, and Cognitive Impairment in Patients with Schizophrenia. *Neuropsychiatric Disease and Treatment*. 17:3597-3606.

44. Masopust J, Maly R, Andrys C, Valis M, Bazant J, Hosak L (2013): The Dynamics of Haemostatic Parameters in Acute Psychotic Patients: A One-Year Prospective Study. *Psychiatria Danubina*. 25:142-148.

45. Mathe AA, Sedvall G, Wiesel FA, Nyback H (1980): Increased Content of Immunoreactive Prostaglandin-E in Cerebrospinal-Fluid of Patients with Schizophrenia. *Lancet*. 1:16-18.

46. Milleit B, Smesny S, Rothermundt M, Preul C, Schroeter ML, von Eiff C, et al. (2016): Serum S100B Protein is Specifically Related to White Matter Changes in Schizophrenia. *Frontiers in Cellular Neuroscience*. 10:14.

47. Mohite S, Yang F, Amin PA, Zunta-Soares G, Colpo GD, Stertz L, et al. (2017): Plasma soluble L-selectin in medicated patients with schizophrenia and healthy controls. *Plos One*. 12:10.

48. Morera-Fumero AL, Diaz-Mesa E, Abreu-Gonzalez P, Fernandez-Lopez L, Cejas-Mendez MD (2017): Day/night changes in serum S100B protein concentrations in acute paranoid schizophrenia. *Progress in Neuro-Psychopharmacology & Biological Psychiatry*. 75:207-212.

49. Morera-Fumero AL, Diaz-Mesa E, Fernandez-Lopez L, Abreu-Gonzalez P, Henry-Benitez MS (2022): Serum s100b protein levels as a neuroinflammatory biomarker of acutely relapsed paranoid schizophrenia patients. *Acta Neuropsychiatr*.1-9.

50. Murphy BP, Pang TY, Hannan AJ, Proffitt TM, McConchie M, Kerr M, et al. (2014): Vascular endothelial growth factor and brain-derived neurotrophic factor in quetiapine treated first-episode psychosis. *Schizophr Res Treatment*. 2014:719395.

51. Neugebauer K, Hammans C, Wensing T, Kumar V, Grodd W, Mevissen L, et al. (2019): Nerve Growth Factor Serum Levels Are Associated With Regional Gray Matter Volume Differences in Schizophrenia Patients. *Front Psychiatry*. 10:275.

52. Nguyen TT, Dev SI, Chen GQ, Liou SC, Martin AS, Irwin MR, et al. (2018): Abnormal levels of vascular endothelial biomarkers in schizophrenia. *European Archives of Psychiatry and Clinical Neuroscience*. 268:849-860.

53. Niitsu T, Ishima T, Yoshida T, Hashimoto T, Matsuzawa D, Shirayama Y, et al. (2014): A positive correlation between serum levels of mature brain-derived neurotrophic factor and negative symptoms in schizophrenia. *Psychiatry Research*. 215:268-273.

54. O'Connell K, Thakore J, Dev KK (2013): Levels of S100B are raised in female patients with schizophrenia. *Bmc Psychiatry*. 13:9.

55. Omori W, Hattori K, Kajitani N, Okada-Tsuchioka M, Boku S, Kunugi H, et al. (2020): Increased Matrix Metalloproteinases in Cerebrospinal Fluids of Patients With Major Depressive Disorder and Schizophrenia. *International Journal of Neuropsychopharmacology*. 23:713-720.

56. Peters J, Van Kammen DP, Gelernter J, Yao J, Shaw D (1990): Neuropeptide Y-like immunoreactivity in schizophrenia. Relationships with clinical measures. *Schizophr Res*. 3:287-294.

57. Pillai A, Howell KR, Ahmed AO, Weinberg D, Allen KM, Bruggemann J, et al. (2016): Association of serum VEGF levels with prefrontal cortex volume in schizophrenia. *Molecular Psychiatry*. 21:686-692.

58. Qi LY, Xiu MH, Chen DC, Wang F, Kosten TA, Kosten TR, et al. (2009): Increased serum S100B levels in chronic schizophrenic patients on long-term clozapine or typical antipsychotics. *Neuroscience Letters*. 462:113-117.

59. Qing Y, Xu LH, Cui GP, Sun LY, Hu XW, Yang XH, et al. (2021): Salivary microbiome profiling reveals a dysbiotic schizophrenia-associated microbiota. *Npj Schizophrenia*. 7:10.

60. Radu G, Luca C, Petrescu L, Bordejevic DA, Mirela TC, Andor M, et al. (2020): The Predictive Value of Endothelial Inflammatory Markers in the Onset of Schizophrenia. *Neuropsychiatric Disease and Treatment*. 16:545-555.

61. Reinikainen KJ, Koponen H, Jolkkonen J, Riekkinen PJ (1990): Decreased Somatostatin-Like Immunoreactivity in the Cerebrospinal-Fluid of Chronic-Schizophrenic Patients with Cognitive Impairment. *Psychiatry Research*. 33:307-312.

62. Roos RP, Davis K, Meltzer HY (1985): Immunoglobulin studies in patients with psychiatric diseases. *Arch Gen Psychiatry*. 42:124-128.

63. Rothermundt M, Missler U, Arolt V, Peters M, Leadbeater J, Wiesmann M, et al. (2001): Increased S100B blood levels in unmedicated and treated schizophrenic patients are correlated with negative symptomatology. *Molecular Psychiatry*. 6:445-449.

64. Rothermundt M, Ponath G, Glaser T, Hetzel G, Arolt V (2004): S100B serum levels and long-term improvement of negative symptoms in patients with schizophrenia. *Neuropsychopharmacology*. 29:1004-1011.

65. Rothermundt M, Ohrmann P, Abel S, Siegmund A, Pedersen A, Ponath G, et al. (2007): Glial cell activation in a subgroup of patients with schizophrenia indicated by increased S100B serum concentrations and elevated myo-inositol. *Progress in Neuro-Psychopharmacology & Biological Psychiatry*. 31:361-364.

66. Rubinow DR (1986): Cerebrospinal fluid somatostatin and psychiatric illness. *Biol Psychiatry*. 21:341-365.

67. Sarandol A, Kirli S, Akkaya C, Altin A, Demirci M, Sarandol E (2007): Oxidative-antioxidative systems and their relation with serum S100B levels in patients with schizophrenia: Effects of short term antipsychotic treatment. *Progress in Neuro-Psychopharmacology & Biological Psychiatry*. 31:1164-1169.

68. Schmitt A, Bertsch T, Henning U, Tost H, Klimke A, Henn FA, et al. (2005): Increased serum S100B in elderly, chronic schizophrenic patients: Negative correlation with deficit symptoms. *Schizophrenia Research*. 80:305-313.

69. Schroeter ML, Abdul-Khaliq H, Fruhauf S, Hohne R, Schick G, Diefenbacher A, et al. (2003): Serum S100B is increased during early treatment with antipsychotics and in deficit schizophrenia. *Schizophrenia Research*. 62:231-236.

70. Schroeter ML, Abdul-Khaliq H, Krebs M, Dieferibacher A, Blasig IE (2009): Neuron-specific enolase is unaltered whereas S100B is elevated in serum of patients with schizophrenia - Original research and meta-analysis. *Psychiatry Research*. 167:66-72.

71. Schwarz MJ, Riedel M, Gruber R, Muller N, Ackenheil M (1998): Autoantibodies against 60-kDa heat shock protein in schizophrenia. *European Archives of Psychiatry and Clinical Neuroscience*. 248:282-288.

72. Seitz-Holland J, Seethaler M, Makris N, Rushmore J, Cho KIK, Rizzoni E, et al. (2022): The association of matrix metalloproteinase 9 (MMP9) with hippocampal volume in schizophrenia: a preliminary MRI study. *Neuropsychopharmacology*. 47:524-530.

73. Severance EG, Gressitt KL, Alaedini A, Rohleder C, Enning F, Bumb JM, et al. (2015): IgG dynamics of dietary antigens point to cerebrospinal fluid barrier or flow dysfunction in first-episode schizophrenia. *Brain Behavior and Immunity*. 44:148-158.

74. Sheikh MA, O'Connell KS, Lekva T, Szabo A, Akkouh IA, Osete JR, et al. (2023): Systemic Cell Adhesion Molecules in Severe Mental Illness: Potential Role of Intercellular CAM-1 in Linking Peripheral and Neuroinflammation. *Biol Psychiatry*. 93:187-196.

75. Shibasaki C, Takebayashi M, Itagaki K, Abe H, Kajitani N, Okada-Tsuchioka M, et al. (2016): Altered Serum Levels of Matrix Metalloproteinase-2,-9 in Response to Electroconvulsive Therapy for Mood Disorders. *International Journal of Neuropsychopharmacology*. 19:8.

76. Smirnova L, Seregin A, Boksha I, Dmitrieva E, Simutkin G, Kornetova E, et al. (2019): The difference in serum proteomes in schizophrenia and bipolar disorder. *Bmc Genomics*. 20:14.

77. Stefanovic MP, Petronijevic N, Dunjic-Kostic B, Velimirovic M, Nikolic T, Jurisic V, et al. (2016): Role of sICAM-1 and sVCAM-1 as biomarkers in early and late stages of schizophrenia. *Journal of Psychiatric Research*. 73:45-52.

78. Steiner J, Bielau H, Bernstein HG, Bogerts B, Wunderlich MT (2006): Increased cerebrospinal fluid and serum levels of S100B in first-onset schizophrenia are not related to a degenerative release of glial fibrillar acidic protein, myelin basic protein and neurone-specific enolase from glia or neurones. *Journal of Neurology Neurosurgery and Psychiatry*. 77:1284-1287.

79. Steiner J, Walter M, Wunderlich MT, Bernstein HG, Panteli B, Brauner M, et al. (2009): A New Pathophysiological Aspect of S100B in Schizophrenia: Potential Regulation of S100B by Its Scavenger Soluble RAGE. *Biological Psychiatry*. 65:1107-1110.

80. Turkmen BA, Yazici E, Erdogan DG, Suda MA, Yazici AB (2021): BDNF, GDNF, NGF and Klotho levels and neurocognitive functions in acute term of schizophrenia. *BMC Psychiatry*. 21:562.

81. Tylec A, Skalecki M, Kocot J, Kurzepa J (2021): Activity of selected metalloproteinases in neurodegenerative diseases of the central nervous system as exemplified by dementia and schizophrenia. *Psychiatria Polska*. 55:1221-1233.

82. Usta A, Kilic F, Demirdas A, Isik U, Doguc DK, Bozkurt M (2021): Serum zonulin and claudin-5 levels in patients with schizophrenia. *European Archives of Psychiatry and Clinical Neuroscience*. 271:767-773.

83. Uzbay T, Goktalay G, Kayir H, Eker SS, Sarandol A, Oral S, et al. (2013): Increased plasma agmatine levels in patients with schizophrenia. *Journal of Psychiatric Research*. 47:1054-1060.

84. Wahlbeck K, Rimón R, Fyhrquist F (1993): Elevated angiotensin-converting enzyme (kininase II) in the cerebrospinal fluid of neuroleptic-treated schizophrenic patients. *Schizophr Res*. 9:77-82.

85. Wahlbeck K, Ahokas A, Nikkilä H, Miettinen K, Rimón R (1997): A longitudinal study of cerebrospinal fluid angiotensin-converting enzyme in neuroleptic-treated schizophrenia. *Prog Neuropsychopharmacol Biol Psychiatry*. 21:591-599.

86. Wahlbeck K, Ahokas A, Miettinen K, Nikkilä H, Rimón R (1998): Higher cerebrospinal fluid angiotensin-converting enzyme levels in neuroleptic-treated than in drug-free patients with schizophrenia. *Schizophr Bull*. 24:391-397.

87. Widerlov E, Lindstrom LH, Wahlestedt C, Ekman R (1988): Neuropeptide-Y and Peptide-Yy as Possible Cerebrospinal-Fluid Markers for Major Depression and Schizophrenia, Respectively. *Journal of Psychiatric Research*. 22:69-79.

88. Wiesmann M, Wandinger KP, Missler U, Eckhoff D, Rothermundt M, Arolt V, et al. (1999): Elevated plasma levels of S-100b protein in schizophrenic patients. *Biol Psychiatry*. 45:1508-1511.

89. Xiao WH, Zhan QQ, Ye F, Tang XW, Li J, Dong H, et al. (2018): Baseline serum vascular endothelial growth factor levels predict treatment response to antipsychotic medication in patients with schizophrenia. *European Neuropsychopharmacology*. 28:603-609.

90. Yazla E, Kayadibi H, Cetin I, Aydinoglu U, Karadere ME (2022): Evaluation of Changes in Peripheric Biomarkers Related to Blood Brain Barrier Damage in Patients with Schizophrenia and Their Correlation with Symptoms. *Clin Psychopharmacol Neurosci*. 20:504-513.

91. Zakharyan R, Atshemyan S, Gevorgyan A, Boyajyan A (2014): Nerve growth factor and its receptor in schizophrenia. *BBA Clin*. 1:24-29.

92. Zhang XY, Xiu MH, Chen DC, Zhu FY, Wu GY, Haile CN, et al. (2010): Increased S100B serum levels in schizophrenic patients with tardive dyskinesia: Association with dyskinetic movements. *Journal of Psychiatric Research*. 44:429-433.

93. Zhang XY, Xiu MH, Song C, Chen DC, Wu GY, Haile CN, et al. (2010): Increased serum S100B in never-medicated and medicated schizophrenic patients. *Journal of Psychiatric Research*. 44:1236-1240.
